# A Novel Feedback Loop That Controls Bimodal Expression of Genetic Competence

**DOI:** 10.1371/journal.pgen.1005047

**Published:** 2015-06-25

**Authors:** Pamela Gamba, Martijs J. Jonker, Leendert W. Hamoen

**Affiliations:** 1 Centre for Bacterial Cell Biology, Institute for Cell and Molecular Biosciences, Newcastle University, Newcastle upon Tyne, United Kingdom; 2 MicroArray Department and Integrative Bioinformatics Unit, Swammerdam Institute for Life Sciences, University of Amsterdam, Amsterdam, The Netherlands; 3 Bacterial Cell Biology, Swammerdam Institute for Life Sciences, University of Amsterdam, Amsterdam, The Netherlands; Indiana University, UNITED STATES

## Abstract

Gene expression can be highly heterogeneous in isogenic cell populations. An extreme type of heterogeneity is the so-called bistable or bimodal expression, whereby a cell can differentiate into two alternative expression states. Stochastic fluctuations of protein levels, also referred to as noise, provide the necessary source of heterogeneity that must be amplified by specific genetic circuits in order to obtain a bimodal response. A classical model of bimodal differentiation is the activation of genetic competence in *Bacillus subtilis*. The competence transcription factor ComK activates transcription of its own gene, and an intricate regulatory network controls the switch to competence and ensures its reversibility. However, it is noise in ComK expression that determines which cells activate the ComK autostimulatory loop and become competent for genetic transformation. Despite its important role in bimodal gene expression, noise remains difficult to investigate due to its inherent stochastic nature. We adapted an artificial autostimulatory loop that bypasses all known ComK regulators to screen for possible factors that affect noise. This led to the identification of a novel protein Kre (YkyB) that controls the bimodal regulation of ComK. Interestingly, Kre appears to modulate the induction of ComK by affecting the stability of *comK* mRNA. The protein influences the expression of many genes, however, Kre is only found in bacteria that contain a ComK homologue and, importantly, *kre* expression itself is downregulated by ComK. The evolutionary significance of this new feedback loop for the reduction of transcriptional noise in *comK* expression is discussed. Our findings show the importance of mRNA stability in bimodal regulation, a factor that requires more attention when studying and modelling this non-deterministic developmental mechanism.

## Author Summary

Gene expression can be highly heterogeneous in clonal cell populations. An extreme type of heterogeneity is the so-called bistable or bimodal expression, whereby a cell can differentiate into two alternative expression states, and consequently a population will be composed of cells that are ‘ON’ and cells that are ‘OFF’. Stochastic fluctuations of protein levels, also referred to as noise, provide the necessary source of heterogeneity that must be amplified by autostimulatory feedback regulation to obtain the bimodal response. A classical model of bistable differentiation is the development of genetic competence in *Bacillus subtilis*. Noise in expression of the transcription factor ComK ultimately determines the fraction of cells that enter the competent state. Due to its intrinsic random nature, noise is difficult to investigate. We adapted an artificial autostimulatory loop that bypasses all known ComK regulators, to screen for possible factors that affect noise in the bimodal regulation of ComK. This led to the discovery of Kre, a novel factor that controls the bimodal expression of ComK. Kre appears to affect the stability of *comK* mRNA. Interestingly, ComK itself represses the expression of *kre*, adding a new double negative feedback loop to the intricate ComK regulation circuit. Our data emphasize that mRNA stability is an important factor in bimodal regulation.

## Introduction

**Fig 1:**
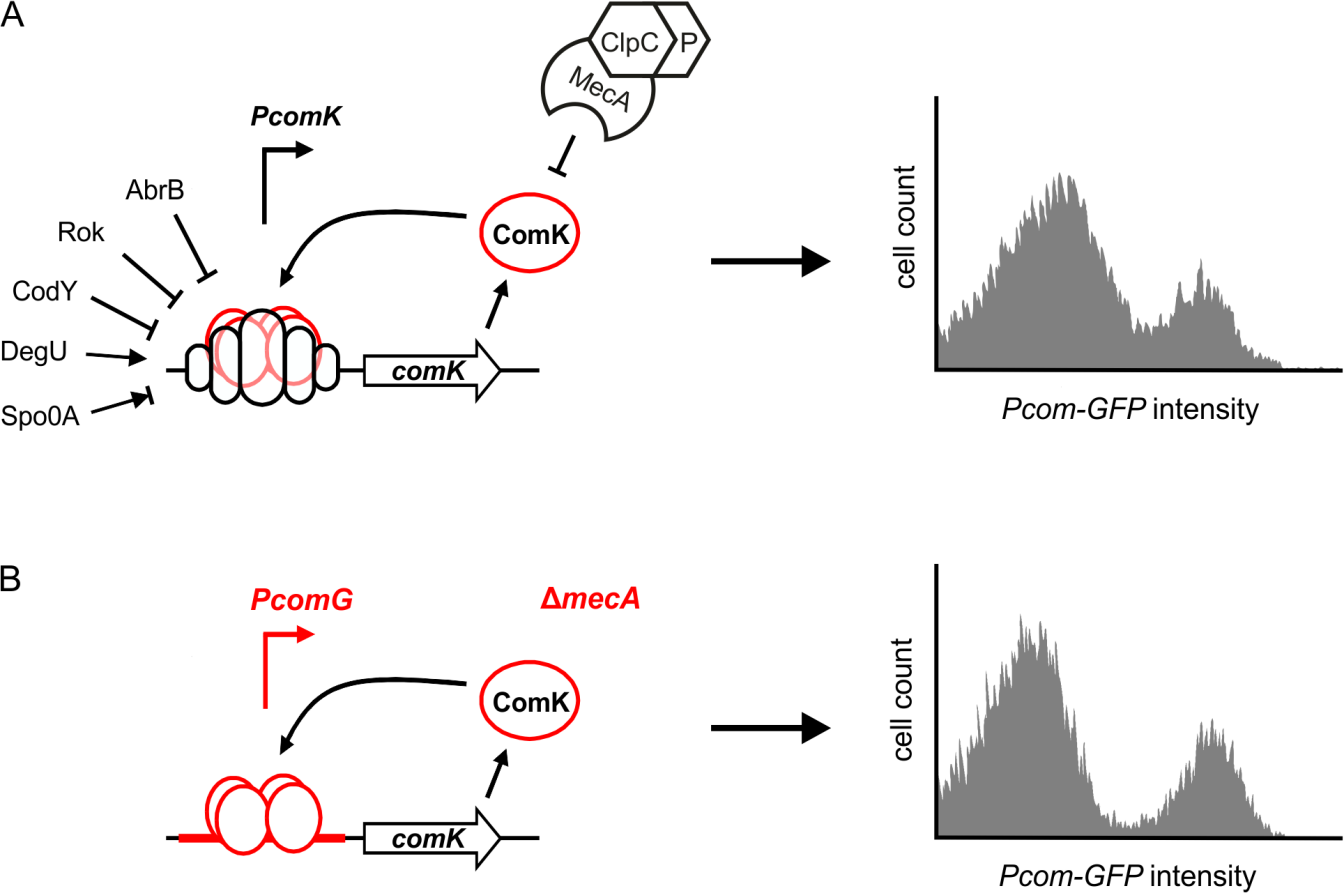
Autostimulation of ComK expression is sufficient for bimodal distribution. (A) Schematic presentation of *comK* regulation in wild-type cells (left panel), and the resulting induction of ComK in a cell population (right panel). (B) Schematic presentation of the artificial ComK feedback loop constructed by replacement of the *comK* promoter with the *comG* promoter and deletion of *mecA*, and the resulting induction of ComK in a cell population (right panel). Induction of competence gene expression (*Pcom-gfp*) was measured after 4 hours in stationary phase using flow cytometry and a GFP reporter fusion (data based on [7]). Binding of transcriptional regulators is indicated by open circles at the promoter region of the gene, and positive or negative action is depicted with arrows or perpendicular lines, respectively. See main text for details.

Cellular differentiation is guided by complex gene regulatory networks that integrate different intra- and extracellular signals. This deterministic view has been challenged by the discovery of so-called bistable or bimodal regulation, whereby the decision to differentiate is stochastic. A classic example is the development of genetic competence in *Bacillus subtilis* [1]. Despite the fact that all cells are genetically identical, and are exposed to the same environmental conditions, only a minor fraction of a *B*. *subtilis* culture will develop into genetically transformable cells. Thus, a competent culture is composed of two different cell types. In essence, this bimodal distribution is the result of the positive feedback loop that regulates expression of the competence transcription factor ComK (Fig 1A) [2]. ComK is responsible for the expression of proteins required for DNA uptake and integration, but it also activates its own transcription [3–6]. If the cellular levels of ComK exceed a certain threshold, the auto-stimulatory loop is triggered and this leads to a rapid accumulation of ComK, which causes entry into the competent state [7–9]. Stochastic fluctuations or ‘noise’ in gene expression ultimately determines which cells accumulate sufficient ComK to reach the threshold level for autoactivation [10].

In recent years it has become apparent that bimodal gene regulation processes are common and occur both in prokaryotic as well as in eukaryotic cells [11,12]. For example, in *B*. *subtilis* the induction of motility, expression of extracellular proteases, and sporulation are bimodal differentiation processes that use positive feedback regulation loops [13–15]. The evolutionary reason for heterogenic differentiation in isogenic cell populations is often explained as a bet-hedging strategy, since bacteria cannot predict how and when environmental conditions will change. However, there are also examples of bimodal differentiation where both cell types benefit from each other. For example, during infection, *Salmonella typhimurium* differentiates into a slow-growing subpopulation expressing virulence genes and a fast-growing subpopulation that is avirulent. However, the latter subpopulation is required to maintain the infection [16]. Bimodal differentiation also occurs in multicellular systems, such as the development of alternative colour vision photoreceptors in *Drosophila melanogaster* [17]. Because of their important role in development, bimodal regulatory feedback loops have been extensively studied and modelled. An intriguing and often debated issue is the role of expression noise in bimodal regulation. The origin of protein expression noise resides in the omnipresent stochastic fluctuations in basic biochemical processes, including transcription, translation, mRNA and protein stability. This noise leads to slight cell-to-cell variations in protein levels [18]. Expression noise is a key prerequisite for bimodal gene expression, and yet noise is an intrinsically stochastic and non-deterministic process. Here, we describe a novel genetic screen that was developed to identify possible cellular factors influencing this noise.

The competence transcription factor ComK is induced in response to nutrient starvation and high cell densities. Entry into the competent state causes severe changes in the physiology of the cell, including a block in growth, cell division and DNA replication [19,20]. An intricate regulatory network ensures that activation of ComK is tightly controlled, and transcription from the *comK* promoter is regulated by five other transcription factors: Rok, AbrB, CodY, DegU and Spo0A [21–25] (Fig 1A). These transcription factors are involved in the regulation of several other differentiation pathways such as sporulation and motility, and they are part of extensive and intertwined regulatory networks [26]. ComK is able to activate *comK* transcription without the necessity to replace the repressors CodY and Rok [27]. Binding of ComK to its own promoter is stimulated by the pleiotropic response regulator DegU [25,28]. Phosphorylated Spo0A also binds to the *comK* promoter region and transiently induces expression by antagonizing Rok [24]. However, increased concentrations of Spo0A repress *comK* transcription, and this master regulator imposes temporal limitations to the onset of competence [24]. Despite the presence of multiple repressors, *comK* is still transcribed at a basal level, and ComK is actively removed by the adaptor protein MecA, which targets it for degradation by the ClpCP protease complex [29]. This proteolytic control is alleviated by a small protein ComS that binds to MecA and prevents ComK degradation [30]. ComS synthesis depends on the production of quorum-sensing pheromones and is therefore cell-density dependent [31,32].

Due to the complexity of *comK* regulation, it seems logical that fluctuations in the different regulation pathways will result in a heterogenic development of competence. However, we have previously shown that only the autostimulation of *comK* expression is sufficient for bimodal expression [7]. This was illustrated by constructing a simplified ComK feedback loop, whereby the *comK* promoter was substituted with the promoter of the *comG* operon (Fig 1B). This operon encodes proteins required for DNA uptake. The *comG* promoter is directly induced by ComK and is not controlled by any other known ComK regulator [33]. Subsequent deletion of *mecA* created an autostimulatory ComK loop that bypasses all known transcriptional and post-translational regulation (Fig 1B). Interestingly, expression of ComK by this artificial ComK feedback loop is comparable to the bimodal ComK expression in a wild type culture (Fig 1B) [7].

The *B*. *subtilis* competence regulation pathway is one of the best studied and modelled natural bimodal developmental systems and is therefore a good model system to study noise in bimodal regulation. We reasoned that the simplified positive feedback loop depicted in Fig 1B provides a way to identify possible unknown cellular factors that influence noise in *comK* expression. If none such factor can be found then we must assume that the bimodal distribution of Fig 1B is solely determined by noise. We developed a mutagenesis screen using the artificial ComK feedback loop of Fig 1B coupled with a reporter construct to visualize its activity. Interestingly, we were able to find transposon mutants that affected the expression of this minimalistic bistable positive feedback loop. Localization of the transposon insertions revealed an unknown gene, *ykyB*, which influences the bimodal induction of ComK. Inactivation of this gene increases the fraction of ComK expressing cells and the gene was therefore renamed *kre* for ComK repressor. Kre has no homology with any other protein. Further analyses indicated that Kre influences the stability of *comK* mRNA. Interestingly, the activity of Kre appears to be more general and is not limited to *comK*, however, the expression of *kre* is specifically downregulated by ComK itself. Kre is only present in species that contain ComK homologues. This co-evolution raises some intriguing questions concerning the balance between benefits and fitness drawbacks of genetic competence in *B*. *subtilis*. Finally, we discuss the importance of regulated RNA degradation in ComK expression and conclude that mRNA stability requires more attention in the research of bimodal gene expression.

## Results

### Identification of Kre (YkyB)

**Fig 2:**
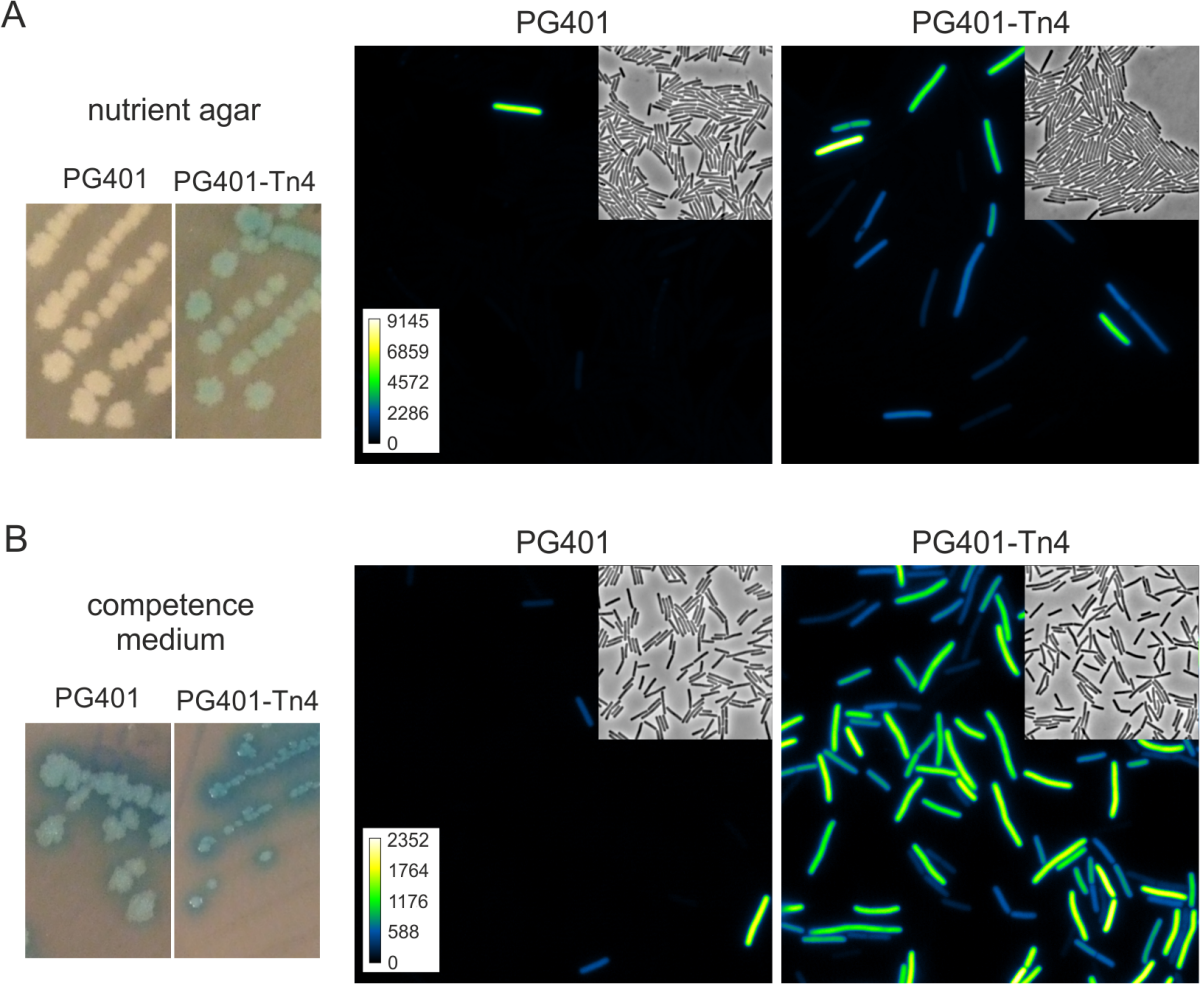
Transposon insertion in *ykyB* increases the activation of an artificial ComK feedback loop. Strains PG401 (*amyE*::*PcomG-lacZ-gfp*, *PcomG-comK*, Δ*mecA*) and PG401-Tn4 (*amyE*::*PcomG-lacZ-gfp*, *PcomG-comK*, Δ*mecA*, *ykyB*:*Tn*) were grown on nutrient agar plates (A) or competence medium plates (B) supplemented with X-gal. Cells from plates were imaged by fluorescent light microscopy. Insets show related phase contrast images and arbitrary GFP colour intensity scales. Pictures and microscopy images were taken after overnight incubation at 37°C.

Previously, we have shown that an artificial autostimulatory ComK feedback loop shows a bimodal expression pattern that closely resembles the wild-type pattern of ComK expression (Fig 1) [7]. Theoretical modelling has shown that a simple positive feedback loop can produce a bimodal response if there is sufficient noise in the expression of the activator and a threshold level for activation [11]. Binding of ComK to DNA is highly cooperative and presumably this non-linear reaction determines the hypersensitive response of the positive feedback loop to small fluctuations of ComK levels [34]. This leaves expression noise as an important determinant of the fraction of ComK expressing cells and therefore of the bimodal distribution. To identify possible factors that influence the bimodal outcome of this artificial feedback loop, we constructed a *lacZ-gfp* operon that is driven by the *comG* promoter. This reporter enables the screening of mutants on plate as well as by fluorescence light microscopy, which makes it possible to distinguish differences in cellular ComK levels from differences in the frequency of ComK expressing cells. The *PcomG-lacZ-gfp* reporter was integrated at the ectopic *amyE* locus and combined with the artificial ComK feedback loop resulting in strain PG401 (*PcomG-comK*, Δ*mecA*, *PcomG-lacZ-gfp*). On nutrient agar plates containing X-gal, PG401 colonies developed a faint blue colour after 2 days of incubation. However, PG401 colonies develop a clear blue colour after overnight incubation on competence medium plates (Fig 2), indicating that medium composition still influences the artificial ComK feedback loop. This is surprising since this loop was constructed in such way that none of the known competence regulators are able to influence its activity (Fig 1). Nevertheless, the effect on nutrient agar plates could be used to our advantage since it facilitates the selection of mutants with different *lacZ*, thus ComK, activities.

Strain PG401 was mutagenized using the *mariner* transposon TnYLB-1 [35], and a library of ~30,000 transposons was plated on nutrient agar plates supplemented with X-gal. Colonies that were blue after one day of incubation were checked for heterogenic GFP expression by microscopy. Four independent insertions were found that mapped in the coding sequence of *ykyB*, a gene of unknown function. Strains with transposon insertions in *ykyB* grew as blue colonies of normal size on nutrient agar plates, and formed dark blue and small colonies when streaked on competence medium plates (Fig 2B). Microscopic visualization revealed that inactivation of *ykyB* causes a strong activation of the artificial ComK feedback loop, with GFP-expressing cells appearing on nutrient agar plates, and developing with more than 4 fold higher frequency on competence medium plates. (Figs 2, S1A and S1B). To confirm that the inactivation of YkyB was responsible for this effect, a complete deletion of *ykyB* was constructed and introduced into the artificial ComK feedback loop strain. When the resulting strain PG539 (*PcomG-comK*, Δ*mecA*, Δ*ykyB*, *PcomG-lacZ-gfp*) was streaked onto competence medium X-gal plates, again dark blue colonies where formed in which more than 80% of cells expressed GFP. A strong activation of the artificial ComK loop was also observed in liquid rich medium (S1C Fig). Since inactivation of *ykyB* causes increased activation of ComK, the gene was renamed Kre for ComK repressor.

### Kre influences competence development

**Fig 3:**
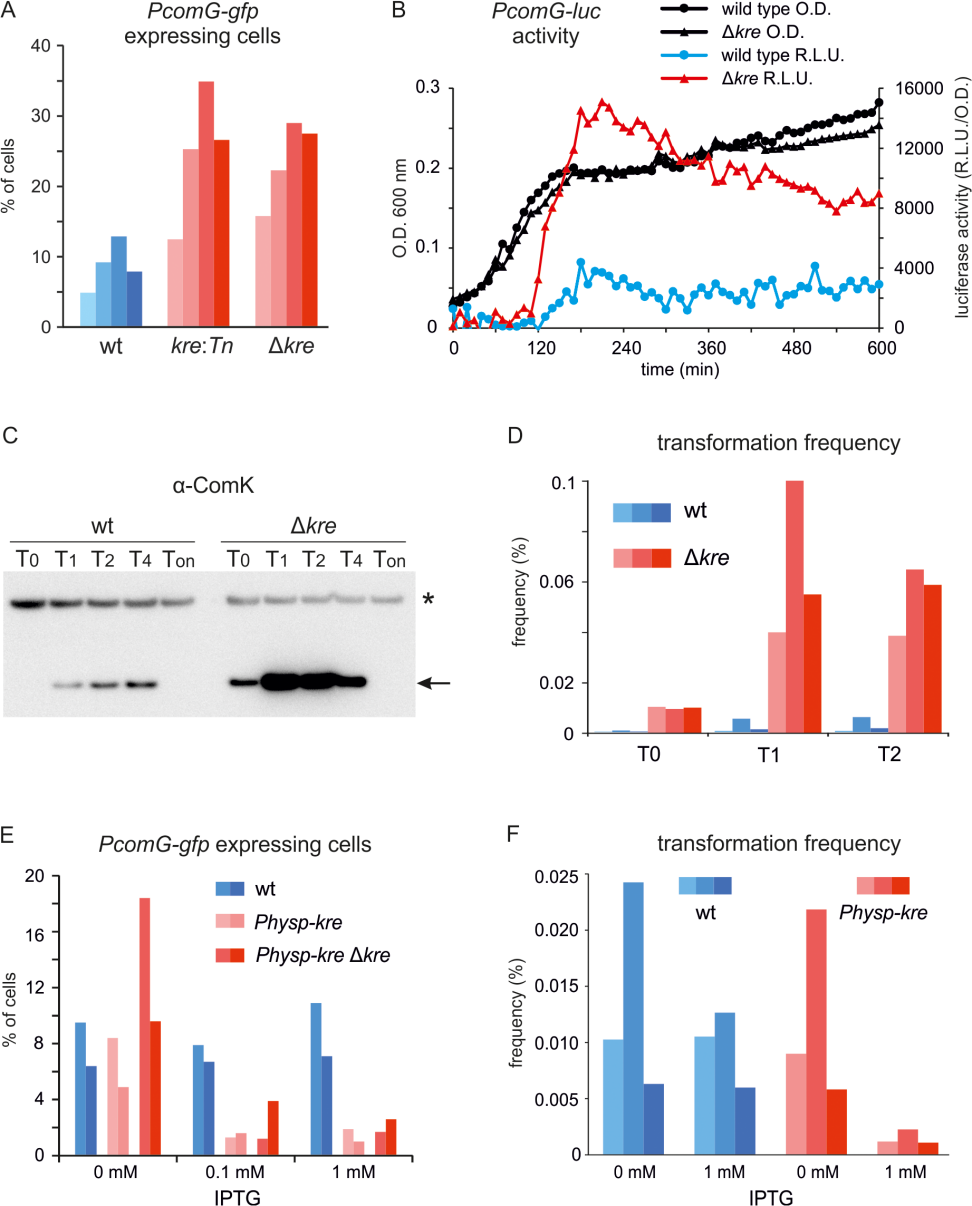
Altered levels of Kre affect competence development in wild-type strains. (A) Fraction of *PcomG* expressing cells in the presence (wt) and absence of *kre* (*kre*:*Tn* and Δ*kre*). Strains PG389 (*amyE*::*PcomG-lacZ-gfp*), PG433 (*amyE*::*PcomG-lacZ-gfp*, *kre*:*Tn*) and PG488 (*amyE*::*PcomG-lacZ-gfp*, Δ*kre*) were grown overnight at 37°C on competence medium plates and GFP levels were measured using fluorescence light microscopy. Cells were counted as *PcomG* ‘ON’ when the GFP intensity exceeded 200 A.U. At least 300 cells were measured for each strain, and the results of 4 independent experiments are shown. (B) Luciferase expression from *PcomG* in wild-type (●) and Δ*kre* mutant (▲) strains. Strains PG710 (*PcomG-luc*) and PG724 (*PcomG-luc*, Δ*kre*) were grown in competence medium at 37°C in a plate reader in the presence of luciferin. Relative luminescence readings and O.D._600_ are plotted. (C) Western blot analysis of ComK levels in wild type (BSB1) and Δ*kre* mutant strain (PG479). Cultures were grown in competence medium at 37°C. Time is given in hours relative to the point of transition to the stationary growth phase (T_0_). T_on_ indicate samples that were taken after prolonged stationary phase growth (overnight incubation). Arrow indicates ComK band and star indicates an aspecific protein band. (D) Transformation frequencies of wild type (wt) strain BSB1 and Δ*kre* mutant strain (PG479) grown in competence medium at 37°C. DNA was added 0, 1 and 2 hours (T0, T1, T2) relative to the point of transition to stationary phase. Transformation frequencies were determined by plating on selective and unselective plates and results of 3 independent experiments are shown. (E) Fraction of *PcomG* expressing cells when *kre* is overexpressed. Strains PG342 (*comG*:*comG-gfp*), PG490 (*comG*:*comG-gfp*, *amyE*::*Physp-kre*) and PG491 (*comG*:*comG-gfp*, *amyE*::*Physp-kre*, Δ*kre*) were grown overnight at 37°C on competence medium plates supplemented with 0, 0.1 or 1 mM IPTG, and the fractions of ‘*PcomG* ON’ cells were determined as in (A). Results of 2 independent experiments are shown. (F) Transformation frequencies when *kre* is overexpressed. Wild type (wt) strain BSB1 and strain PG474 (*amyE*::*Physp-kre*) were grown in competence medium in the presence or absence of 1 mM IPTG and transformed using a two-step starvation protocol used for routine transformations. Results of 3 independent experiments are shown.

To test whether inactivation of *kre* also influences ComK induction in wild type cells, both the *kre*:*Tn* mutation as well as the Δ*kre* deletion were introduced into a wild-type background containing the *PcomG-lacZ-gfp* reporter fusion (strains PG433 (*amyE*::*PcomG-lacZ-gfp*, *kre*:*Tn*) and PG488 (*amyE*::*PcomG-lacZ-gfp*, Δ*kre*)). The resulting strains showed an approximately 3 fold increase in the number of GFP expressing cells when grown overnight on competence medium plates (Fig 3A), indicating that the effect of a *kre* mutation is observable in wild type cells, and is not limited to strains containing the artificial ComK feedback loop. Similar results were obtained when the GFP reporter was fused to promoters of the competence genes *comC*, *comF*, *addAB* and *nucA* (S2 Fig). This shows that the sensitivity for Kre is not a unique property of the *comG* promoter, and that Kre affects ComK activity. To test the effect of a *kre* deletion in liquid cultures, we made use of the sensitive luciferase reporter fusion [36]. As shown in Fig 3B, a clear induction of the *PcomG-luc* reporter fusion is observed when *kre* is deleted. ComK levels were then checked by Western blotting and, as shown in Fig 3C, a strong increase in the intensity of ComK bands was detected for the *kre* mutant compared to the wild type strain. Consistently, when tested under the same growth conditions, an approximately 30-fold increase in transformation frequency of a *kre* mutant was observed at 0, 1 and 2 hours after the transition to stationary phase (Fig 3D).

The results so far suggest that *kre* encodes a negative regulator of ComK. To confirm this, *kre* was placed under control of the strong IPTG-inducible *Phyper-spank* promoter at the ectopic *amyE* locus [37]. Indeed, overexpression of Kre reduced the fraction of *PcomG-gfp* expressing cells approximately 5 fold, and a strong repression was observed even when the wild type *kre* allele was deleted (Fig 3E). Overexpression of Kre also reduced the transformation efficiency (Fig 3F). To confirm that the effect was due to the Kre protein, a frame-shift mutation in the start codon of *kre* was introduced. The resulting strain (PG548) was unaffected by the addition of IPTG and showed normal transformation efficiencies (S3 Fig).

### Kre is a cytosolic protein of unknown function

*kre* encodes a hypothetical protein of 154 amino acids with no homology to any known protein. A recent comprehensive transcriptome analysis revealed that *kre* is expressed as a monocistronic mRNA (S4 Fig) at moderate levels in different growth conditions [38]. In this analysis, slightly higher expression levels were observed in M9 medium, as well as under salt, ethanol, and heat stress, and no major difference were observed between exponential growth and stationary growth, at least in rich medium [38].

Possibly, Kre functions as a transcription factor and regulates ComK expression, although no DNA binding or any other conserved motifs are apparent from its amino acid sequence. Many transcription factors bind to the nucleoid, owing to their DNA binding property [39]. To examine whether Kre co-localizes with the nucleoid, GFP fusions to the N- and C-terminal ends of the protein were constructed (S5 Fig). Overexpression of both GFP-fusions reduced the fraction of *PcomG* expressing cells, indicating that the fusions are at least partially functional (S6 Fig). However, both fusions showed a diffuse cytoplasmic GFP signal (S5 Fig), suggesting that the protein does not function as a simple DNA binding transcription factor.

### Kre activity is not *comK* locus dependent

Recent transcriptome experiments revealed the presence of a counter transcript, S365 RNA, which overlaps with the *comK* gene and is transcribed from the downstream located *yhxD* gene [38] (S7 Fig). YhxD is strongly upregulated under stress conditions such as the presence of high salt, ethanol or high temperatures, conditions that also result in some increase in *kre* expression [38]. Possibly, the induction of *yhxD* is regulated by Kre and the anti-sense S365 transcript interferes with *comK* expression. To test this, the *PcomG-comK* construct was relocated from the *comK* locus to the ectopic *aprE* locus, and the wild type *comK* gene was replaced with a phleomycin resistance cassette. The resulting strain (PG461; *aprE*::*PcomG-comK*, Δ*mecA*, Δ*comK*, *amyE*::*PcomG-lacZ-gfp*) showed GFP expression comparable to PG401 (S7 Fig). Introduction of the *kre* mutation into PG461 resulted in an increase in GFP expressing cells very similar to what was observed in previous experiments with strains that contain the *PcomG-comK* construct at the wild-type *comK* locus (Figs 2 and S7). Thus, the activity of Kre is not based on the induction of the S365 anti-sense transcript.

### Post-transcriptional control

**Fig 4:**
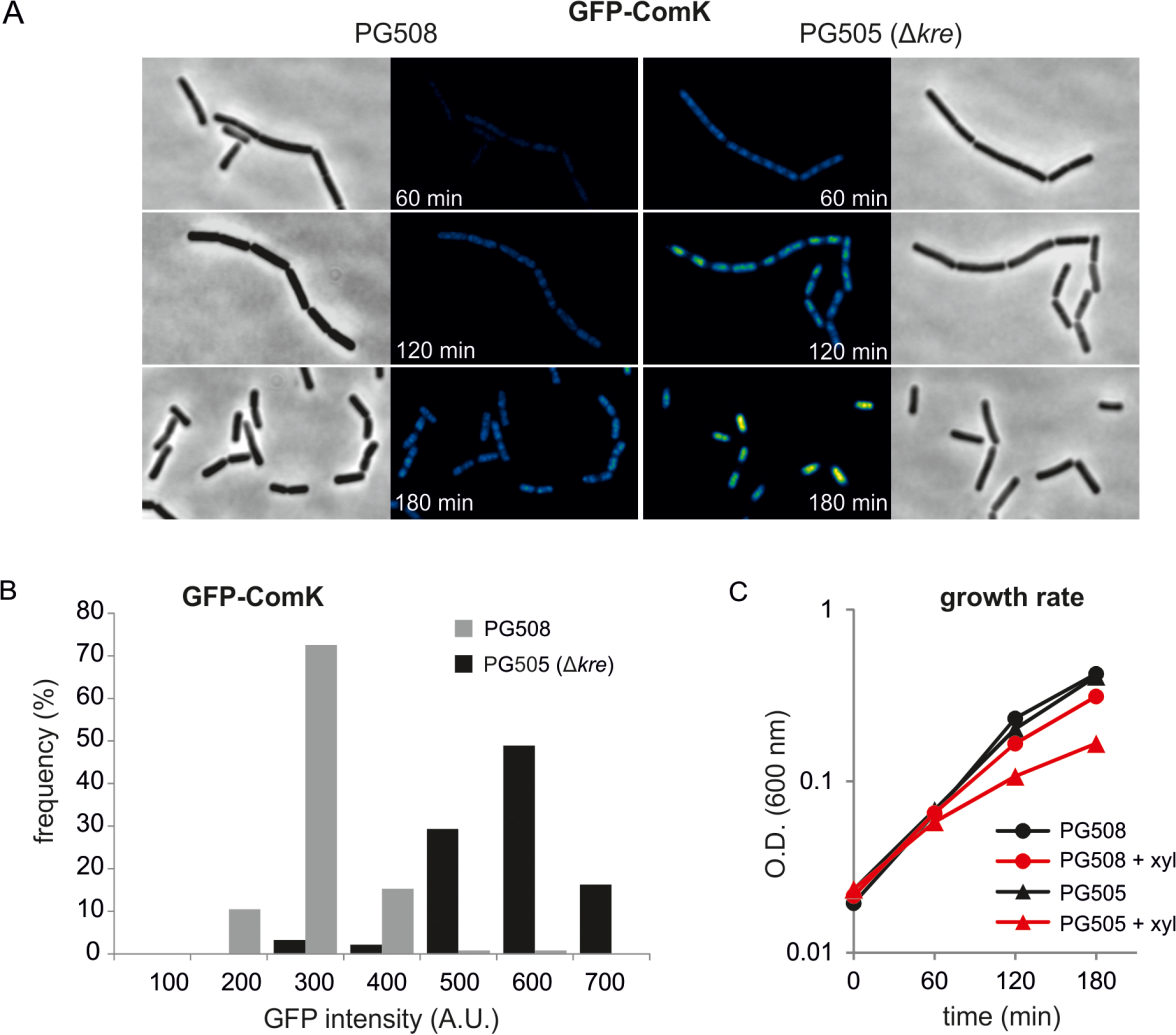
Absence of Kre increases xylose induced GFP-ComK expression. (A) GFP and phase contrast images of strains PG508 (*amyE*::*Pxyl-gfp-comK*, Δ*comK*, Δ*mecA*) and PG505 (*amyE*::*Pxyl-gfp-comK*, Δ*comK*, Δ*mecA*, *kre*:*Tn*) 60, 120 and 180 min after induction of GFP-ComK with 0.05% xylose. Fluorescence levels are indicated by a colour intensity scale using the same contrast settings. (B) Quantification of GFP-ComK levels after 60 min of xylose induction. (C) Induction of GFP-ComK causes a stronger reduction in cell growth when Kre is inactivated.

So far, we have tested the effect of a *kre* mutation in the presence of ComK autostimulation. To further dissect at which level Kre controls the bimodal induction of ComK, we uncoupled ComK expression from its autostimulatory transcription by removing the native *comK* gene and by placing a copy under control of the xylose inducible *Pxyl* promoter at the ectopic *amyE* locus. To monitor the effect on ComK, the protein was N-terminally fused to GFP. The *mecA* gene was also deleted to prevent possible proteolytic regulation effects. Since the GFP-ComK translational fusion is partially active and binds to DNA, a clear fluorescent nucleoid signal is observed (Fig 4A). Interestingly, when a *kre* mutation was introduced into the strain, the fluorescence signal increased significantly (Fig 4A and 4B). The increase in GFP-ComK expression also resulted in a further reduction in growth rate (Fig 4C). Since the *kre* deletion has an effect on GFP-ComK accumulation even in the absence of a *comK* promoter, we conclude that Kre is not directly regulating the capacity of ComK to activate its own promoter.

### Kre activity is not restricted to *comK*

**Fig 5:**
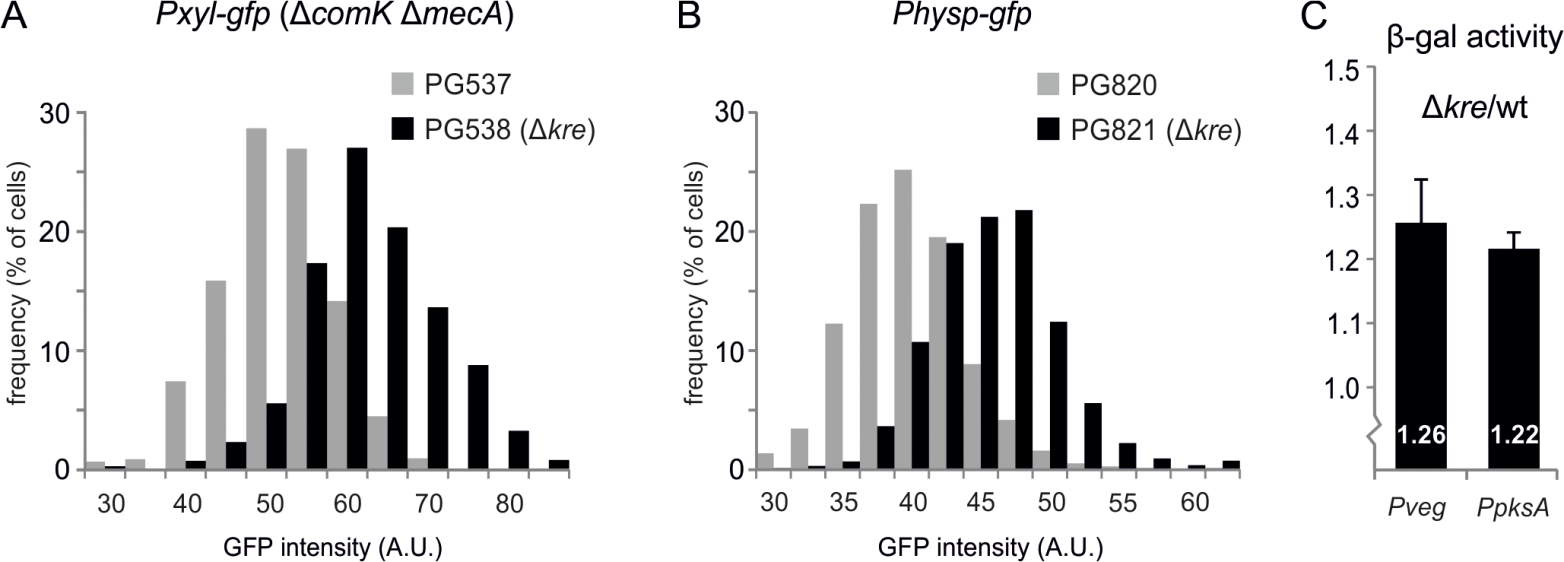
General effect of Kre on gene expression. (A) Absence of *kre* leads to increased GFP expression from a *Pxyl* promoter. Strains PG537 (*amyE*::*Pxyl-gfp*, Δ*comK*, Δ*mecA*) and PG538 (*amyE*::*Pxyl-gfp*, Δ*comK*, Δ*mecA kre*:*Tn*) were grown to logarithmic phase in LB at 37°C in the presence of 0.1% xylose. Graph shows the results of one representative experiment (3 biological replicates). (B) Absence of *kre* leads to increased GFP expression from the *Physp* promoter in a wild type background. Strains PG820 (*amyE*::*Physp-gfp*) and PG821 (*Physp-gfp*, *kre*:*Tn*) were grown to logarithmic phase in LB at 37°C in the presence of 50 μM IPTG. Graph shows the results of one representative experiment (3 biological replicates). (C) Increase in β-galactosidase expression when *kre* is deleted. PG500 (*amyE*::*Pveg-lacZ-gfp*), PG512 (*amyE*::*Pveg-lacZ-gfp*, Δ*kre*), PG811 (*amyE*::*PpksA-lacZ-gfp*), PG815 (*amyE*::*PpksA-lacZ-gfp*, Δ*kre*) were grown in LB at 37°C and samples were collected at O.D._600_ ~0.2–0.3 for β-galactosidase activity measurements. Graphs show the ratio between *kre* mutant and wild-type strain averaged over 3 independent experiments.

As a negative control for the experiments of Fig 4, the fluorescence levels in a strain that expresses GFP instead of ComK-GFP were measured. Surprisingly, it appeared that the introduction of a *kre* deletion in this strain also resulted in increased GFP expression levels (Fig 5A). To examine whether this effect might be linked to the *Pxyl* promoter or to the *mecA comK* double mutant background that was used, the *kre* mutation was introduced into a wild type background strain containing an IPTG inducible *Physp-gfp* reporter fusion (strain PG820). As shown in Fig 5B, also this promoter produced higher levels of GFP when *kre* was mutated. Finally, to determine whether the Kre activity might be specific for GFP, we tested another reporter and used the β-galactosidase expressing *lacZ-gfp* operon. This time the reporter was driven by the *Pveg* promoter, which is assumed to be unregulated during logarithmic growth [40]. When the *Pveg-lacZ* fusion was measured in a *kre* mutant background (strain PG512), a modest but significant increase in β-galactosidase levels was detected (Fig 5C). Another promoter, *PpksA*, which also seemed to be unregulated according to a recent comprehensive transcriptome study [38], was tested as well and gave a similar increase in expression (Fig 5C). These results suggest that Kre functions as a more general repressor of gene expression.

### Kre transcriptome

To determine the genome wide expression effect of a *kre* mutation, a micro-array experiment was performed. The transcriptome analysis was executed with samples taken from logarithmic growing cultures in rich LB medium. These conditions repress competence development [26], and were chosen to prevent induction of competence genes that might mask indicative gene regulation events. A table of the 68 most relevant affected genes, i.e. genes whose expression difference was more than 4-fold with an adjusted P-value <0.05, is presented in Table 1. The list comprises a mixture of metabolic genes, genes involved in iron uptake, as well as several genes with unknown activities. Two of the genes (*ssbB* and *dprA*) are part of the ComK regulon. However, the list of genes does not reveal a clear regulation pathway that could explain the mechanism of Kre activity.

**Table 1:** Transcriptome analysis of a kre mutant (PG479). Genes are listed with 4-fold expression differences between wild type and a *kre* deletion mutant (PG479). Genes with adjusted p-values for the expression difference larger than 0.05 are discarded. ComK-induced genes are highlighted in bold.

***gene***	**fold down**	**adj. P-val**	**function**
*kre*	88.2	0.001	*comK* repressor
*nrgA*	5.2	0.015	ammonium transporter
*ygzA*	4.8	0.016	hypothetical protein
*yvsH*	4.5	0.043	putative lysine transporter
*yqhR*	4.3	0.038	hypothetical protein
*gin*	4.2	0.015	inhibitor of SigG and SigE
*yfhI*	4.1	0.015	hypothetical protein
***gene***	**fold up**	**adj. P-val**	**function**
*dhbA*	15.1	0.027	biosynthesis of siderophore bacillibactin
*dhbC*	14.5	0.029	biosynthesis of siderophore bacillibactin
*dhbE*	13.8	0.029	biosynthesis of siderophore bacillibactin
*dhbB*	12.4	0.034	biosynthesis of siderophore bacillibactin
*ykuO*	11.0	0.029	hypothetical protein
*ykuP*	10.8	0.033	flavodoxin
*ysbB*	10.2	0.024	hypothetical protein
*licA*	9.8	0.030	lichenan uptake and phosphorylation
*dhbF*	9.7	0.038	biosynthesis of siderophore bacillibactin
*ysbA*	9.3	0.023	putative anti-holin
*ykuN*	9.2	0.028	flavodoxin
*licH*	8.9	0.036	phospho-beta glucosidase (lichenan utilization)
*licC*	8.0	0.033	lichenan uptake and phosphorylation
*rbsA*	6.9	0.016	ribose ABC transporter
*licB*	6.9	0.032	lichenan uptake and phosphorylation
*rbsB*	6.9	0.023	ribose ABC transporter
*rbsC*	6.9	0.020	ribose ABC transporter
*levD*	6.9	0.031	fructose uptake and phosphorylation
*yhaR*	6.8	0.023	hypothetical protein
*besA*	6.6	0.028	trilactone hydrolase (iron acquisition)
*sunT*	6.5	0.012	sublancin lantibiotic ABC transporter
*citZ*	6.4	0.012	citrate synthase
*rocG*	6.0	0.016	glutamate dehydrogenase (arginine utilization)
*levF*	5.9	0.031	fructose uptake and phosphorylation
*rbsD*	5.9	0.017	ribose ABC transporter
*kbl*	5.9	0.018	amino-ketobutyrate CoA ligase (threonine utilization)
*abn2*	5.7	0.015	arabinan degradation
*bdbA*	5.5	0.014	thiol-disulfide oxidoreductase
*levG*	5.3	0.033	fructose uptake and phosphorylation
*ywsB*	5.2	0.033	survival of ethanol and salt stresses
*rbsK*	5.1	0.014	ribokinase (ribose utilization)
*abnA*	5.1	0.021	arabinan degradation
*phrA*	5.1	0.012	phosphatase (RapA) inhibitor
***ssbB***	5.1	0.014	single-strand DNA-binding protein (ComK induced)
*tdh*	5.0	0.020	threonine dehydrogenase (threonine utilization)
*gltP*	4.9	0.029	similar to H+/glutamate symporter
*araP*	4.9	0.014	arabinose ABC transporter
*sunS*	4.8	0.015	biosynthesis of antimicrobial peptide sublancin
*ywcE*	4.7	0.024	holin required for spore morphogenesis germination
*rocA*	4.7	0.017	arginine, ornithine and citrulline utilization
*rbsR*	4.7	0.016	regulation of ribose utilization
*dctP*	4.7	0.029	uptake succinate, fumurate, malate and oxaloacetate
*rapA*	4.6	0.012	response regulator aspartate phosphatase
*xynB*	4.6	0.023	xylan beta-xylosidase
*yobO*	4.6	0.016	hypothetical protein
*levE*	4.5	0.034	fructose uptake and phosphorylation
*ybbJ*	4.5	0.016	hypothetical protein
*sunA*	4.4	0.018	sublancin lantibiotic antimicrobial precursor peptide
*yxeB*	4.4	0.024	hydroxamate siderophore ABC transporter
*xynP*	4.4	0.029	beta-xyloside permease (xylan utilization)
*murQ*	4.4	0.016	cell wall turnover
*ybaR*	4.4	0.012	hypothetical protein
*ald*	4.3	0.020	alanine dehydrogenase (alanine utilization)
*yesL*	4.3	0.020	hypothetical protein
*btr*	4.3	0.015	regulation of iron acquisition
*araN*	4.1	0.016	arabinose ABC transporter
*ybxI*	4.1	0.022	beta-lactamase
*murR*	4.1	0.016	probably regulation of muramic acid utilization
*phrK*	4.1	0.020	phosphatase (RapK) regulator
***dprA***	4.0	0.018	recombination mediator protein (ComK induced)
*gcvPA*	4.0	0.016	glycine dehydrogenase (glycine utilization)

### Kre affects *comK* mRNA stability

**Fig 6:**
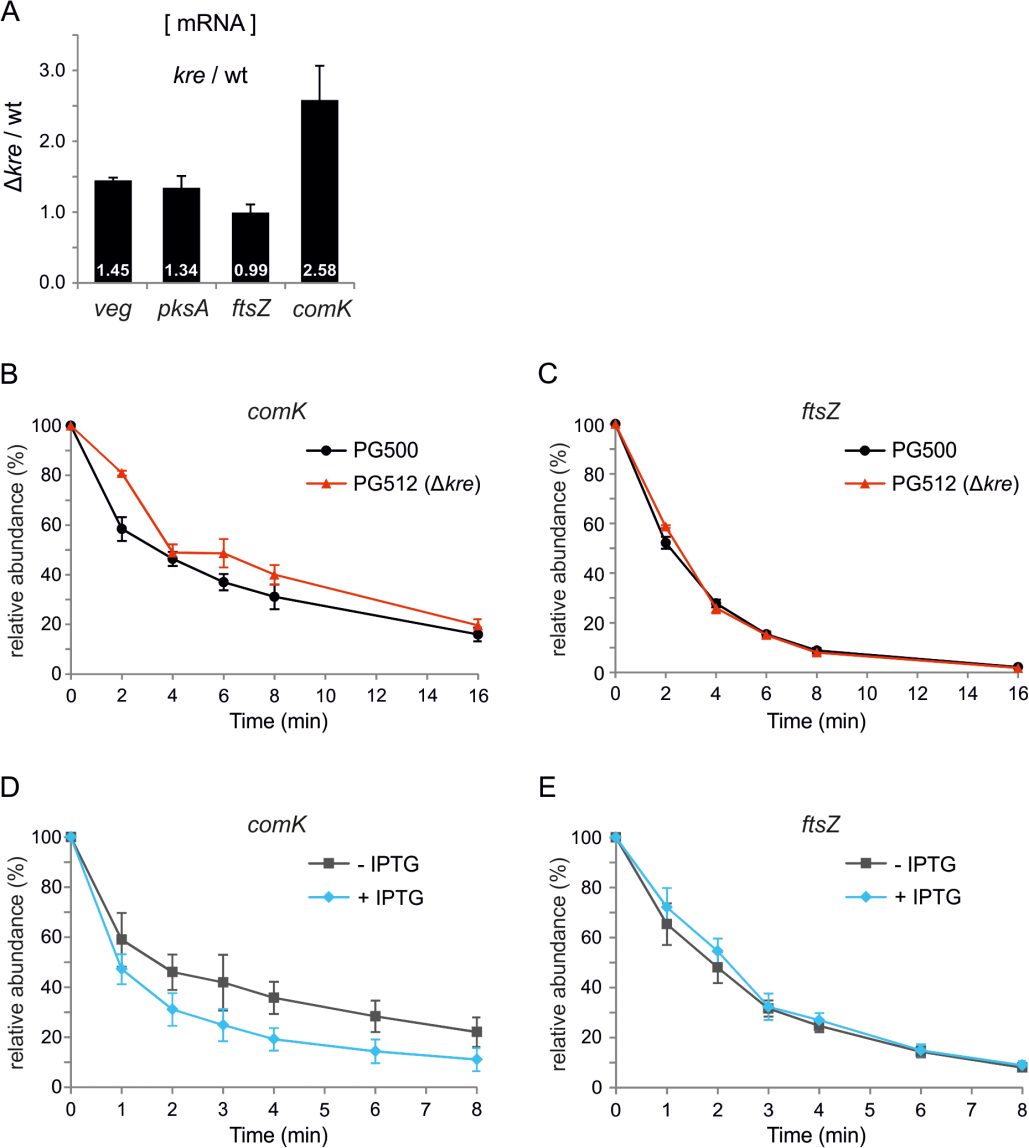
Kre affects *comK* mRNA stability. (A) Relative increase in *veg*, *pksA* and *comK* mRNA levels in a *kre* mutant determined by quantitative real-time PCR (qPCR). RNA was isolated from PG500 (*amyE*::*Pveg-lacZ-gfp*) and PG512 (*amyE*::*Pveg-lacZ-gfp*, Δ*kre*), and results shown are the average of 3 biological replicates. (B & C) Strains PG500 (*amyE*::*Pveg-lacZ-gfp*) and PG512 (*amyE*::*Pveg-lacZ-gfp*, Δ*kre*) were grown in LB at 37°C. At OD_600_ ~0.2 T0-samples were collected immediately before rifampicin (150 μg/ml) was added. Subsequent samples were taken 2, 4, 6, 8 and 16 min after rifampicin addition. Relative abundance of *comK* (B) and *ftsZ* (C) transcripts were quantified over 3 independent experiments using qPCR. (D & E) Strain PG474 (*amyE*::*Physp*-*kre*) was grown in LB at 37°C in the presence or absence of 1 mM IPTG. At OD_600_ ~0.25, T0-samples were collected immediately before rifampicin was added. Subsequent samples were taken 1, 2, 3, 4, 6, and 8 minutes after rifampicin addition. Relative abundance of *comK* (D) and *ftsZ* (E) transcripts were quantified over 3 independent experiments using qPCR.

Kre reduces the expression of different unrelated genes but it is unclear whether this control occurs at the transcriptional or translational level. Therefore, we determined the levels of the *veg*, *pksA* and *comK* transcripts using qPCR. As shown in Fig 6A, the *veg* and *pksA* mRNA levels are higher in a *kre* mutant background, associated with p-values of 0.002 and 0.06, respectively. The effect on *comK* mRNA is the strongest (Fig 6A, p-value 0.013), which is presumably a consequence of the autostimulatory transcription of this gene. Not all genes are upregulated when Kre is deleted, as is apparent from the transcriptome data (Table 1), and a qPCR experiment showed that the mRNA levels of the cell division gene *ftsZ* are unaffected in a *kre* mutant strain (Fig 6A, p-value 0.88). These and previous data suggest that Kre is not a general inhibitor of RNA polymerase or protein translation, but that the protein affects mRNA levels and possibly influences mRNA stability. To test this, *comK* mRNA levels were measured after addition of the RNA polymerase inhibitor rifampicin. In the absence of Kre, an increase in stability was detected, with the half-life increasing on average from 3.9 min (SE = 0.4 min) to 5.4 min (SE = 0.6 min). Such increase was consistently observed in three biological replicates (Fig 6B and S1 Table). Comparing these 3 independent replicate measurements at each time point using a statistical test, showed that the increase in stability was significant (false discovery rate corrected p-value ≤ 0.05, S1 Table). As a control, we measured the stability of *ftsZ* mRNA, but there was no apparent effect when Kre was absent (Fig 6C, mRNA half-life of ~2.2 min in both strains, and S1 Table). We then measured the stability of the same transcripts upon Kre overexpression by using a strain containing an extra copy of *kre* under control of the strong IPTG inducible *Phyperspank* promoter. As shown in Fig 6D, a significant decrease in stability was detected for *comK* mRNA (false discovery rate corrected p-value ≤ 0.05, S1 Table) while, again, the stability of *ftsZ* mRNA was unaffected (Fig 6E). The half-life of *comK* mRNA was ~2.6 min in the absence, and ~1.3 min in the presence of IPTG, respectively (S1 Table). We note that, even in the absence of inducer, the half-life of *comK* mRNA was shorter compared to a wild type background. This might be due to leakiness of the *Phyperspank* promoter, and suggests that small variations of Kre levels may be sufficient to alter *comK* levels. Under the same conditions, the half-life of the *ftsZ* transcript was ~1.9 min and ~2.1 min, respectively. Based on these data, we conclude that Kre controls the bimodal response of ComK induction by affecting the stability of *comK* mRNA. The effect of Kre appears modest. However, the autostimulatory feedback will amplify small variations.

### *kre* is repressed in competent cells

**Fig 7:**
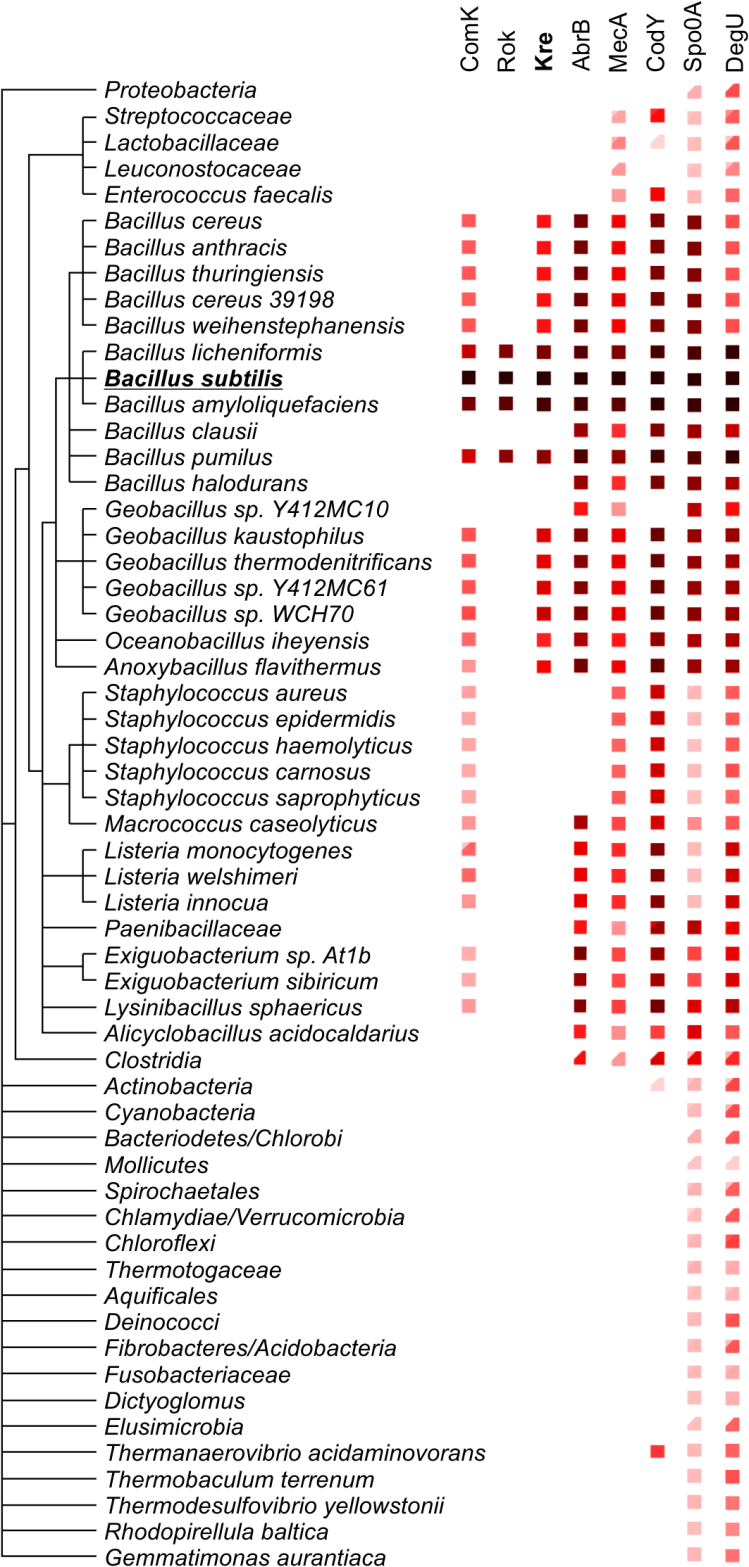
Phylogenetic relation between *comK* and its key regulators. Phylogenetic display of *kre* and other ComK regulators in bacterial species. Data and presentation is based on information from the STRING interaction database [75]. Colour intensity indicates measure of homology with the corresponding genes in *B*. *subtilis*.

**Fig 8:**
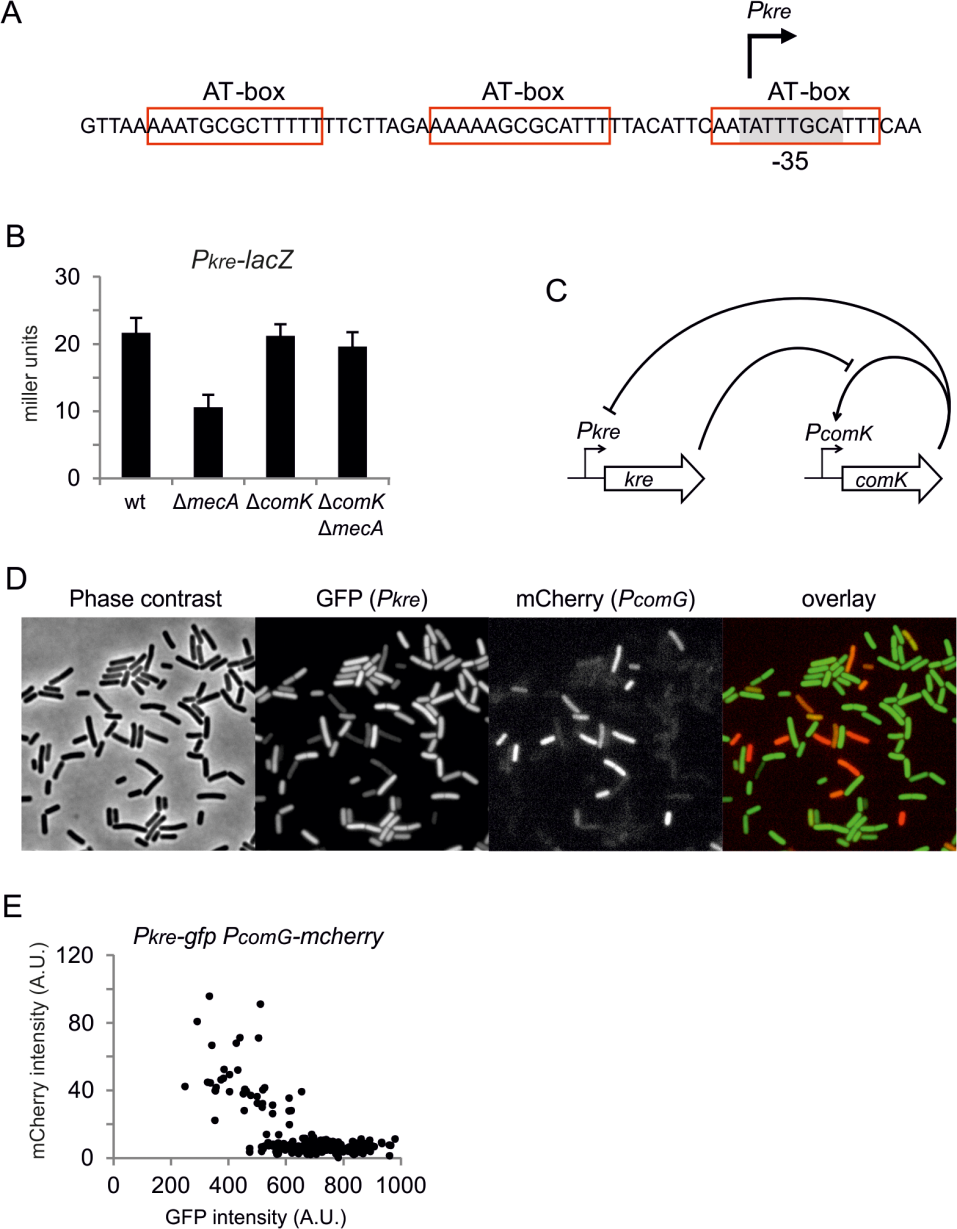
Negative feedback regulation of *kre*. (A) Schematic representation of the *kre* promoter region. Three potential AT-boxes and the putative -35 promoter region [38] are highlighted in red and grey, respectively. (B) Overproduction of ComK in a *mecA* mutant causes repression of *kre* expression. Strains PG501 (*amyE*::*Pkre-lacZ-gfp*), PG763 (*amyE*::*Pkre-lacZ-gfp*, Δ*mecA*), PG764 (*amyE*::*Pkre-lacZ-gfp*, Δ*comK*) and PG765 (*amyE*::*Pkre-lacZ-gfp*, Δ*mecA*, Δ*comK*) were grown in competence medium at 37°C, and samples were collected at OD_600_ ~0.1 for β-galactosidase measurements. (C) Schematic representation of the double negative feedback regulation exerted by Kre and ComK. (D) Reciprocal correlation between *Pkre* and *PcomG* expression. Strain PG688 (*kre*:*Pkre-gfp*, *amyE*::*PcomG-mcherry*) was grown in competence medium at 37°C, and phase contracts and fluorescent images were taken after overnight incubation. (E) Average GFP and mCherry levels in single cells from the same culture in D (n = 211).

Kre affects the expression of many genes, yet there is a significant ‘presence-absence’ correlation between *kre* and *comK* in different bacterial genomes (Fig 7). A closer inspection of the *kre* promoter revealed the presence of at least 3 potential ComK dimer binding sites (Fig 8A). These so-called AT-boxes are spaced by 8 nucleotides, which is the correct distance to allow for the strong binding of a ComK tetramer [34]. Thus the *kre* promoter contains at least two ComK binding sites, one of which overlaps with the RNA polymerase binding site (-35 region). To examine whether ComK influences *kre* promoter activity, a *Pkre*-*lacZ-gfp* reporter fusion was cloned into a *mecA* deletion strain, which overproduces ComK due to the absence of the regulatory proteolytic control of ComK [41]. As shown in Fig 8B, deletion of *mecA* decreases the β-galactosidase activity by half, and this reduction was ComK dependent. The fact that overproduction of ComK suppresses this promoter implies a new negative feedback loop in the control of ComK expression in *B*. *subtilis* (Fig 8C). To examine whether this feedback control occurs in wild type cells expressing normal levels of ComK, we measured the activity of both the *kre* and *comG* promoters in single cells. The latter promoter was used as a reporter for ComK expression. The promoter of *kre* was fused to GFP by means of a Campbell-type integration (*kre*:*Pkre*-*gfp)*, and the *comG* promoter was fused to mCherry and cloned into the *amyE* locus (*amyE*::*PcomG*-*mcherry*). Fluorescence light microscopy images of cells from a competent culture showed a clear reciprocal staining in the green and red channels (Fig 8D). Quantification of the fluorescent signals indicated that *comG* expressing cells show on average a 60% reduction in the *Pkre*-GFP signal (Fig 8E). The heterogenic expression of *Pkre*-GFP disappeared in a *comK* mutant strain (S8 Fig). Thus, the negative feedback control of *kre* is active in wild type cells. When *kre* was placed under control of the *PcomG* promoter, and therefore activated by ComK instead of repressed, a strong reduction in transformation efficiency was observed (S9 Fig). We conclude that, even though Kre affects the expression of many genes, its activity is closely intertwined with the development of genetic competence in *B*. *subtilis*.

## Discussion

### Novel ComK control pathway

Stochastic fluctuations in protein expression are a key prerequisite for the bimodal activation of positive feedback regulation systems. These random fluctuations in gene regulation pathways are often compared to the ‘noise’ in electronic circuits. The way electrical noise in circuits can be dampened, so can random spikes in protein levels be dampened too. This has consequences for bimodal processes, since a decrease in the amplitude or frequency of these spikes will reduce the chance that an activator reaches the threshold level necessary for auto-activation. Peaks in stochastic protein expression can be moderated by (i) suppressing the chance of transcription, (ii) reducing the life time (stability) of the mRNA, (iii) suppressing the chance of translation, and (iv) reducing the life time of the protein. The first and fourth mechanism are well known control pathways in the bimodal induction of ComK: Transcription from the *comK* promoter is repressed by 4 different transcription factors (Rok, AbrB, CodY and Spo0A), and the adaptor protein MecA stimulates degradation of ComK by the ClpCP protease complex. The identification of Kre reveals the presence of a third mechanism: control of *comK* mRNA stability.

### Evolution of noise control

The complexity of ComK regulation remains puzzling, especially since bimodal expression can be obtained without the necessity of an intricate regulation network (Fig 1B). However, there are two main reasons why additional regulation is required, the timing of competence development and the escape from the competence state [26]. The latter is achieved by proteolytic degradation of ComK due to the reactivation of MecA as a consequence of dwindling ComS levels late in stationary phase [29,42]. Proper timing of ComK expression is essential since competent cells do not grow. Therefore, this developmental process should only be induced when nutrients become limiting. This explains for example the control of *comK* by the metabolic regulator CodY and the transition state regulator AbrB [22,23]. Competence induction should also not occur when cells are sporulating, therefore the control by the key sporulation activator Spo0A [24]. However, the reasons for the regulation by Rok and Kre are not immediately apparent. What is interesting is that these proteins were acquired relatively recently in evolutionary terms (Fig 7). Possibly, the origin of Rok and Kre regulation resides in a high fitness burden of the competence state (competent cells do not grow) relative to fitness benefits. In fact, there is only a remote chance that a genetic competent cell will acquire genetic material from which it can immediately benefit. In this respect, it is important to realize that most wild *B*. *subtilis* isolates are poorly competent, at least under laboratory conditions, and only the domesticated and mutagenized *B*. *subtilis* 168 strain shows high levels of competence [43]. Presumably, most of the time it is better for cells to circumvent the induction of competence, and acquiring new repressors that reduce the fraction of competent cell, like Rok and Kre, might therefore be beneficial. However, in the long term, the capacity to obtain new genetic material benefits the species, and this might explain why the negative feedback regulation of *kre* by ComK has evolved. Related to this, it is maybe interesting to note that the expression of *rok* is also repressed by ComK [21]. Of course, we cannot rule out that the main function of Kre is to restrict the time cells stay in the dormant genetic competent state.

### RNA regulation

Our data suggests that Kre regulates mRNA stability. The protein does not have a known RNA or nucleotide binding pocket and its activity seems to be more general and not restricted to *comK* transcripts. It is unclear by which mechanism Kre influences mRNA decay. Within the ComK regulation cascade there is one other pathway that is affected by RNA modification. The conserved exoribonuclease PnpA, which is involved in cellular RNA homeostasis [44], is required for the expression of ComS [45]. The small *comS* gene is embedded within a very long (~26kb) mRNA encoding the synthetase subunits for the lipopeptide antibiotic surfactin [31,32]. Why PnpA is required for the expression of ComS is not known. Interestingly, in our screen we found two mutations that repressed the artificial ComK autostimulatory loop. These mutants contained transposon insertions into *pnpA* and *cshA*. The latter gene encodes a conserved RNA helicase which is also involved in cellular RNA homeostasis [46]. Thus, it seems there are more factors influencing *comK* mRNA stability. In a recent study it was shown that in *Halobacterium salinarum* and *Escherichia coli* there are specific RNases that control transcriptional positive autoregulation loops involved in certain energy-related processes [47]. Clearly, regulation of mRNA life-time is an efficient and presumably common mechanism to control transcriptional positive feedback loops.

### Growth and medium regulation

Two features of the artificial bimodal ComK loop remain unexplained. Even when *kre* is deleted, the induction of ComK is still growth phase and medium dependent (Figs 2 and 3). One explanation for the growth phase dependent expression is that ComK expressing cells are unable to divide. However, preliminary time lapse microscopy experiments showed a strong induction in the number of ComK expressing cells after the logarithmic growth phase has ceased. A more plausible explanation is that the exponential increase in cell volume during logarithmic growth dilutes any ComK that is expressed, and only when growth slows down will ComK accumulate to levels necessary to pass the threshold level required for auto-activation [48].

Optimal induction of competence in *B*. *subtilis* occurs in minimal medium with glucose as energy source. In contrast to this, in rich Luria Broth (LB) medium almost no competent cells can be detected [49]. Surprisingly, activation of the artificial ComK feedback loop is still medium dependent and clearly more efficient in minimal competence medium rather than in rich medium, even when *kre* is absent (Fig 2). One key difference between LB and minimal competence medium is the presence of glucose. Interestingly, when glucose was added to LB medium, there was a substantial increase in cells activating the artificial bimodal ComK loop (S2 Table). There is an intriguing link between glycolysis and the cellular RNA processing and degradation machinery [50,51]. The core of the RNA degradosome in *B*. *subtilis* exists of the essential endoribonuclease RNase Y that forms a complex with other RNases, including PnpA, and the RNA helicase CshA. Importantly, the glycolytic enzymes enolase and phosphofructokinase are also part of this large protein complex [52]. This interaction is found in many other bacterial species [53]. It is as yet unknown how these glycolytic enzymes influence the RNA degradosome activity, but it might provide a clue for the glucose dependent regulation of competence.

Here, we have described a new level of regulation of a well-known bimodal developmental pathway. Further research is required to elucidate the molecular mechanism of action of Kre and to identify its functional partners. However, it is clear that the role of mRNA stability in noise control may play a more significant role than previously appreciated.

## Materials and Methods

### Bacterial strains and growth conditions

Strains and plasmids used in this study are listed in Table 2. All the *B*. *subtilis* strains were derivatives of BSB1, a tryptophan-prototrophic (*trp* ^+^) derivative of the 168 *trpC2* strain [38]. *B*. *subtilis* strains were grown at 30°C or 37°C on Nutrient agar plates (Oxoid), or in liquid LB or competence medium (78 mM K_2_HPO_4_, 42.8 mM KH_2_PO_4_, 14.7 mM (NH_4_)_2_SO_4_, 6.6 mM MgSO_4_, 3.3 mM Na_3_-citrate, 26.9 mM glucose, 95 μM tryptophan, 4.2 μM ferric ammonium citrate, 0.02% Casamino acids). Solid competence medium was prepared with 1.5% Purified Agar (Oxoid) and was supplemented with a mixture of 13 amino acids (Gly, Asn, Val, Glu, Leu, Asp, Ile, Pro, Phe, Ser, Ala, Thr, Gln) at a final concentration of 10 μg/ml each to improve growth. To relieve catabolite repression of the *Pxyl* promoter in competence medium, glucose was replaced by fructose [54]. For selection, nutrient agar plates were supplemented with 10 μg/ml tetracycline, 5 μg/ml chloramphenicol, 50 μg/ml spectinomycin, 5 μg/ml kanamycin, 1 μg/ml phleomycin or 0.5 μg/ml erythromycin together with 25 μg/ml lincomycin. Xylose and IPTG were used as inducers at concentrations of 0.05–0.1% and 50 μM-1 mM respectively. *E*. *coli* was used as cloning intermediate.

**Table 2:** Strains and plasmids used in this study. Unless stated otherwise, all strains were made in the BSB1 wild type background [38]. Genes responsible for resistance to antibiotics are abbreviated as follows: *bla* (ampicillin), *cat* (chloramphenicol), *erm* (erythromycin), *kan* (kanamycin), *phleo* (phleomycin), *spc* (spectinomycin), *tet* (tetracycline).

**Strain**	**Relevant features or genotype**	**Construction, source or reference**
***B*. *subtilis***		
BSB1	*trp+*	[38]
AH7	*amyE*::*Physp-gfp spc*	A. Henderson
BV2096	*mecA*::*kan PcomG-comK spc*, *comG-gfp cat*	[7]
H121G	*trpC2 amyE*::*Pxyl-gfp-comK spc*, *comK*::*kan*	H. Strahl
PG341	*PcomG-comK spc*	BV2096 DNA → BSB1
PG342	*comG-gfp cat*	BV2096 DNA → BSB1
PG343	*mecA*::*kan*	BV2096 DNA → BSB1
PG349	*amyE*::*Pxyl-gfp-comK spc*	H121G → BSB1
PG368	*mecA*::*tet*	This study
PG389	*amyE*::*PcomG-lacZ-gfp+ cat*	pPG40 → BSB1
PG401	*amyE*::*PcomG-lacZ-gfp+ cat*, *PcomG-comK spc*, *mecA*::*tet*	[PG341+PG368] DNAs → PG389
PG401-Tn4	*amyE*::*PcomG-lacZ-gfp+ cat*, *PcomG-comK spc*, *mecA*::*tet*, *kre*:TnYLB1#4	This study
PG405	*amyE*::*PcomG-mcherry spc*	pPG38 → BSB1
PG406	*kre*:TnYLB1#4 *kan*	PG401-Tn4 DNA → BSB1
PG425	*comG-gfp cat*, *kre*:TnYLB1#4 *kan*	PG401-Tn4 DNA → PG342
PG433	*amyE*::*PcomG-lacZ-gfp+ cat*, *kre*:TnYLB1#4	PG401-Tn4 DNA → PG389
PG435	*amyE*::*PcomC-lacZ-gfp+ cat*	pPG55 → BSB1
PG436	*amyE*::*PcomF-lacZ-gfp+ cat*	pPG56 → BSB1
PG437	*amyE*::*PaddAB-lacZ-gfp+ cat*	pPG57 → BSB1
PG438	*amyE*::*PnucA-lacZ-gfp+ cat*	pPG58 → BSB1
PG442	*amyE*::*Pxyl-msfGFP-kre spc*	pPG54 → BSB1
PG445	*amyE*::*Pxyl-mGFPmut1 spc*	pHJS103 → BSB1
PG447	*comK*::*phleo*	This study
PG448	*amyE*::*PcomC-lacZ-gfp+ cat*, *kre*:TnYLB1#4 *kan*	PG401-Tn4 DNA → PG435
PG449	*amyE*::*PcomF-lacZ-gfp+ cat*, *kre*:TnYLB1#4 *kan*	PG401-Tn4 DNA → PG436
PG450	*amyE*::*PaddAB-lacZ-gfp+ cat*, *kre*:TnYLB1 #4 *kan*	PG401-Tn4 DNA → PG437
PG455	*aprE*::*PcomG-comK spc*	This study
PG457	*amyE*::*PnucA-lacZ-gfp+ cat*, *kre*:TnYLB1#4 kan	PG401-Tn4 DNA → PG438
PG458	*aprE*::*PcomG-comK spc*, *amyE*::*PcomG-lacZ-gfp*	PG455 DNA → PG389
PG459	*aprE*::*PcomG-comK spc*, *amyE*::*PcomG-lacZ-gfp*, *kre*:TnYLB1#4 *kan*	PG455 DNA → PG433
PG461	*aprE*::*PcomG-comK spc*, *mecA*::*tet*, *comK*::*phleo*, *amyE*::*PcomG-lacZ-gfp cat*	PG447 and PG368 DNAs → PG458
PG463	*aprE*::*PcomG-comK spc*, *mecA*::*tet*, *comK*::*phleo*, *amyE*::*PcomG-lacZ-gfp cat*, *kre*:TnYLB1#4 *kan*	PG447 and PG368 DNAs → PG459
PG474	*amyE*::*Physp-kre spc*	pPG59 → BSB1
PG475	*amyE*::*Pxyl-gfp-comK spc*, *kre*:TnYLB1#4 *kan*	PG406 DNA → PG349
PG477	*amyE*::*Pxyl-msfGFP-kre spc*, *kre*:TnYLB1#4 *kan*	PG406 DNA → PG442
PG479	*kre*::*erm*	This study
PG482	*amyE*::*Pxyl-msfGFP-kre spc*, *comG-gfp cat*	PG342 DNA → PG442
PG485	*amyE*::*Pxyl-msfGFP-kre spc*, *kre*:TnYLB1#4 *kan*, *comG-gfp cat*	PG342 → PG477
PG488	*amyE*::*PcomG-lacZ-gfp+ cat*, *kre*::*erm*	PG479 DNA → PG389
PG490	*comG*:*PcomG-gfp cat*, *amyE*::*Physp-kre spc*	PG474 DNA → PG342
PG491	*comG*:*PcomG-gfp cat*, *amyE*::*Physp-kre spc*, *kre*::*erm*	[PG474+PG479] DNAs → PG342
PG494	*amyE*::*Pxyl-kre-mSFgfp*	pPG61 → BSB1
PG500	*amyE*:: *cat Pveg-lacZ-gfp+*	pPG60 → BSB1
PG501	*amyE*:: *cat Pkre-lacZ-gfp+*	pPG62 → BSB1
PG512	*amyE*:: *cat Pveg-lacZ-gfp+*, *kre*::*erm*	PG479 DNA → PG500
PG505	*amyE*::*Pxyl-gfp-comK spc; kre*:TnYLB1#*4 kan*, *comK*::*phleo*, *mecA*::*tet*	[PG447+PG368] DNAs → PG475
PG508	*amyE*::*Pxyl-gfp-comK spc kan*, *comK*::*phleo*, *mecA*::*tet*	[PG447+PG368] DNAs → PG349
PG535	*amyE*::*Pxyl-mGFPmut1 spc*, *kre*:*TnYLB1#4 kan*	PG406 DNA → PG445
PG537	*amyE*::*Pxyl-mGFPmut1 spc*, *comK*::*phleo*, *mecA*::*tet*	[PG447+PG368] DNAs → PG445
PG538	*amyE*::*Pxyl-mGFPmut1 spc*, *kre*:TnYLB1#*4 kan*, *comK*::*phleo*, *mecA*::*tet*	[PG447+PG368] DNAs → PG535
PG548	*amyE*::Physp-kre*	pPG59*fs → BSB1
PG604	*kre*:*Pkre-gfpmut1 cat Pxyl-Pkre-kre*	pPG66 → BSB1 (Campbell)
PG678	*kre*:*Pkre-gfpmut1 cat Pxyl-Pkre-kre*, *comK*::*phleo*	PG447 DNA → PG604
PG688	*kre*:*Pkre-gfpmut1 cat Pxyl-Pkre-kre*, *amyE*::*PcomG-mcherry spc*	PG405 DNA → PG604
PG710	*PcomG*: *cat PcomG-luc+*	pPG118 → BSB1 (Campbell)
PG724	*PcomG*: *cat PcomG-luc+*, *kre*::*erm*	PG710 DNA → PG479
PG746	*aprE*::*PcomG-lacZ-gfp+ kan*	pPG63 → BSB1
PG747	*amyE*::*PcomG-kre spc*	pPG126 → BSB1
PG753	*aprE*::*PcomG-lacZ-gfp+ kan*, *amyE*::*PcomG-kre spc*	PG747 DNA → PG746
PG755	*aprE*::*PcomG-lacZ-gfp+ kan*, *amyE*::*PcomG-kre spc*, *kre*::*erm*	PG479 DNA → PG746
PG763	*amyE*:: *cat Pkre-lacZ-gfp+*, *mecA*::*kan*	PG343 DNA → PG501
PG764	*amyE*:: *cat Pkre-lacZ-gfp+*, *comK*::*phleo*	PG447 DNA → PG501
PG765	*amyE*:: *cat Pkre-lacZ-gfp+*, *mecA*::*kan*, *comK*::*phleo*	[PG343+PG447] DNAs → PG501
PG811	*amyE*::*PpksA-lacZ-gfp+ cat*	pPG136 → BSB1
PG815	*amyE*::*PpksA-lacZ-gfp+ cat*, *kre*::*erm*	PG479 DNA → PG811
PG820	*amyE*::*Physp-gfp spc*	AH7 DNA → BSB1
PG821	*amyE*::*Physp-gfp spc*, *kre*::*erm*	AH7 DNA → PG479
***E*. *coli***		
DH5α	*F-*, *φ80lacZΔM15*, Δ*(lacZYAargF)U196*, *recA1*, *endA1*, *hsdR17*, *(rK-*, *mK+)*, *phoA*, *supE44*, *λ-*, *thi-1*, *gyrA96*, *relA1*	Laboratory stock
**Plasmid**	**Relevant features or genotype**	**Construction, source or reference**
pDR111	*bla*, *amyE3'*, *spc*, *lacI*, *Physp*, *amyE5'*	[37]
pMarB	*bla erm P*_*ctc*_ *Himar1 kan* (TnYLB-1)	[35]
pSG1164	*bla*, *cat*, *Pxyl-gfpmut1*	[61]
pMutin-GFP+	*bla erm Pspac-gfp+ lacI*	[55]
pUC18Cm::luc	*bla cat luc+*	[36]
pAWC3	*bla*, *aprE5'*, *kan*, *lacI*, *lacZ-gfp+ aprE3'*	Gamba *et al*., in preparation
pHJS103	*bla*, *amyE3'*, *spc*, *Pxyl-mgfpmut1*	[61] and H. Strahl
pHJS105	*bla*, *amyE3'*, *spc*, *Pxyl-mSFgfp*, *amyE5'*	[61] and H. Strahl
pPG2	*bla*, *amyE3'*, *spc*, *gfpmut1*, *amyE5'*	[56]
pPG20	*bla*, *erm*, *lacI*, *gfp+*	This study
pPG21	*bla*, *erm*, *lacI*, *mcherry*	This study
pPG22	*bla*, *amyE3'*, *spc*, *gfp+*, *amyE5'*	This study
pPG23	*bla*, *amyE3'*, *spc*, *mcherry*, *amyE5'*	This study
pPG34	*bla*, *amyE3'*, *spc*, *PcomG-gfp+*, *amyE5'*	This study
pPG35	*bla*, *amyE3'*, *spc*, *PcomG-lacZ-gfp+*, *amyE5'*	This study
pPG38	*bla*, *amyE3'*, *spc*, *PcomG-mcherry*, *amyE5'*	This study
pPG40	*bla*, *amyE3'*, *cat*, *PcomG-lacZ-gfp+*, *amyE5'*	This study
pPG49	*bla*, *amyE3'*, *spc*, *mSFgfp*, *amyE5'*	This study
pPG54	*bla*, *amyE3'*, *spc*, *Pxyl-mSFgfp-kre*, *amyE5'*	This study
pPG55	*bla*, *amyE3'*, *cat*, *PcomC-lacZ-gfp+*, *amyE5'*	This study
pPG56	*bla*, *amyE3'*, *cat*, *PcomF-lacZ-gfp+*, *amyE5'*	This study
pPG57	*bla*, *amyE3'*, *cat*, *PaddAB-lacZ-gfp+*, *amyE5'*	This study
pPG58	*bla*, *amyE3'*, *cat*, *PnucA-lacZ-gfp+*, *amyE5'*	This study
pPG59	*bla*, *amyE3'*, *spc*, *lacI*, *Physp-kre amyE5'*	This study
pPG59*fs	*bla*, *amyE3'*, *spc*, *lacI*, Physp-kre*fs amyE5'	This study
pPG60	*bla*, *amyE3'*, *cat*, *Pveg-lacZ-gfp+*, *amyE5'*	This study
pPG61	*bla*, *amyE3'*, *spc*, *Pxyl-kre-msfGFP*, *amyE5'*	This study
pPG62	*bla*, *amyE3'*, *cat*, *Pkre-lacZ-gfp+*, *amyE5'*	This study
pPG63	*bla*, *aprE5'*, *kan*, *lacI*, *PcomG-lacZ- gfp+*, *aprE3'*	This study
pPG66	*bla Pxyl-Pkre-gfpmut1 cat*	This study
pPG118	*bla cat PcomG-luc+*	This study
pPG126	*bla*, *amyE3'*, *spc*, *PcomG-kre*, *amyE5'*	This study
pPG136	*bla*, *amyE3'*, *cat*, *PpksA-lacZ-gfp+*, *amyE5'*	This study

### Construction of plasmids

Molecular cloning, PCRs and *E*. *coli* transformations were carried out using standard techniques. Oligonucleotides used in this study are listed in S3 Table. Plasmids pPG22 and pPG23 were used to construct promoter-*gfp* and promoter-*mcherry* fusions, respectively, at the *amyE* locus. Plasmid pMutin-GFP+ [55] contains a *gfp* reporter with three terminators (t1, t2, t0 from the *rrnB* operon of *E*. *coli*) upstream the multiple cloning site in front of *gfp* and a *trpA* terminator downstream of *gfp*. pMutin-GFP+ was amplified with primers PG187 and PG188 in order to remove the *Pspac* promoter and to introduce 5 unique restriction sites in the multiple cloning site (*Age*I, *Bgl*II, *Pml*I, *Bln*I, *Sac*II). Digestion with *Pml*I and subsequent self-ligation resulted in plasmid pPG20. The *gfp* region with terminators was amplified from pPG20 with primers PG195 and PG196, digested with *Apa*I and *Not*I and ligated into a similarly cut *amyE*-integration vector pPG2 [56], obtaining plasmid pPG22. To construct plasmid pPG23, the *mcherry* gene from plasmid pHM232 [57], was amplified with primers PG189 and PG190, digested with *Eag*I and *Spe*I and inserted into pPG20, obtaining pPG21. The *mcherry* region with terminators was amplified from pPG21 with primers PG195 and PG196 and, after digestion, ligated into pPG2, obtaining pPG23.

*comG* promoter reporters were constructed by amplifying the *comG* promoter region with primers PG201 and PG202 and genomic DNA of strain 168 as template. PCR fragments were digested with *Bgl*II and *Bln*I and ligated into digested pPG22 or pPG23, resulting in plasmids pPG34 and pPG38, respectively. To construct the *lacZ-gfp+* operon reporter for transposon screening, the *lacZ* sequence was amplified from pMutin4 [58] with primers PG203 and PG204, digested with *Sac*II and *Kpn*I and ligated to a similarly cut pPG34, resulting in plasmid pPG35. Plasmid pPG40 was derived from pPG35 by replacing the spectinomycin resistance marker with the chloramphenicol resistance cassette *cat* from pSG1186 [59], which was amplified with primers PG209 and PG210, digested with *Sph*I and *Xma*I and ligated to pPG35. To integrate a *PcomG*-*lacZ-gfp+* reporter at the *aprE* genomic locus, plasmid pPG63 was constructed by amplifying the *comG* promoter with primers PG330 and PG202, and ligating it to pAWC3, a plasmid based on pAPNC213 [60] that carries the *lacZ-gfp+* operon (Gamba *et al*., in preparation), after digestion with *Xba*I and *Bln*I. For the luciferase reporter fusion (*PcomG-luc+*), plasmid pPG118 was constructed by amplifying the *comG* promoter with primers PG418 and PG419, subsequent digestion with *Hind*III and *Bam*HI and ligation into pUC18Cm::luc [36].

Inducible GFP fusions of Kre to msfGFP (monomeric superfolder GFP) were made at the N-terminal or C-terminal end of the protein by cloning the coding sequence of *kre* into plasmids pHJS105 and pPG49, respectively. To make an N-terminal fusion, *kre* was amplified with primers PG287 and PG288, digested with *Bam*HI and *Eco*RI, and ligated to pHJS105, resulting in plasmid pPG54. To make a C-terminal fusion, plasmid pPG49 was first constructed by amplifying *mSFgfp* from pHJS105 with primers PG279 and PG280, digesting the fragment with *Sac*II and *Spe*I, and ligating it to the gel-extracted backbone of pPG22. Then, *Pxyl* promoter and *kre* coding sequences were introduced at the same time into pPG49 with a double ligation step. *Pxyl* was amplified with primers PG320 and PG321 from pSG1729 and digested with *Bgl*II-*Bln*I, while the *kre* gene was amplified with primers PG319 and PG282 and digested *Bln*I-*Sac*II. The two fragments were ligated to a *Bgl*II-*Sac*II cut pPG49, obtaining plasmid pPG61.

Reporter fusions with the promoters of other competence genes were made by amplifying the promoter regions of *comC*, *comF*, *addAB* and *nucA* with primer pairs PG289-PG290, PG291-PG292, PG293-PG294, PG295-PG296, respectively. After digestion with *Bgl*II and *Bln*I the promoter regions were ligated into pPG40, resulting in plasmids pPG55, pPG56, pPG57 and pPG58 respectively.

For overexpression of Kre, *kre* was cloned behind the strong IPTG inducible hyperspank promoter (*Physp*). The *kre* coding sequence was amplified with primers PG299 and PG300, digested with *Sal*I and *Sph*I and ligated into pDR111 [37], resulting in plasmid pPG59. Plasmid pPG59*fs is a variant of pPG59 with a mutation in the ATG start codon of the *kre* coding sequence, which becomes ATAG. This plasmid was obtained by amplifying plasmid pPG59 with oligonucleotides PG332-PG333, which carry the desired mutation. To create a *Pkre*-*lacZ*-*gfp* reporter fusion, the promoter region of *kre* was amplified with primers PG322 and PG323 and, after digestion with *Age*I and *Bln*I, ligated into pPG40 from which the *PcomG* promoter region had been removed by extraction of the cut plasmid form agarose gel, resulting in plasmid pPG62. Plasmid pPG66 was made to create a *Pkre-gfp* promoter fusion at the *kre* locus by means of homologous Campbell-type integration. The plasmid was constructed by amplifying the promoter region of *kre* and its ribosome binding site with primers PG334 and PG336, digested with *Kpn*I and *Pst*I and ligation into pSG1164 [61]. To create a *kre* gene under expression of *PcomG*, plasmid pPG34 was digested with *Bln*I and *Spe*I and gel extracted to remove the *gfp* fragment. The plasmid backbone was then ligated with the *kre* gene that was amplified with PG319 and PG438 from genomic template DNA, resulting in plasmid pPG126.

*lacZ*-reporter fusions with *veg* and *pksA* promoters were made as follow. The promoter region of *veg*, was amplified using primers PG317 and PG318 and, after digestion with *Bgl*II and *Bgl*I, ligated into pPG40 from which the *PcomG* promoter fragment was removed by extraction of the cut plasmid form agarose gel. The resulting plasmid was labelled pPG60. The promoter region of *pksA* was amplified with primers PG509 and PG510, digested with *Bgl*II and *Bgl*I and ligated into pPG60 from which the *Pveg* promoter fragment was removed by extraction of the cut plasmid form agarose gel. The resulting plasmid was named pPG136.

### Construction of *B*. *subtilis* strains

To construct strain PG368 (*mecA*::*tet*), 2.5 kb regions upstream and downstream of *mecA* were amplified with primer pairs PG223-PG224 and PG225-PG226, and subsequently digested with *Bln*I or *Xho*I respectively. A tetracycline resistance cassette was amplified from pBEST309 [62] with primer pairs PG221-PG222, digested with *Bln*I and *Xho*I and ligated to the digested upstream and downstream amplified fragments. Competent *B*. *subtilis* cells were transformed directly with the ligation products and mutants were verified with PCR. To construct strain PG447 (*comK*::*phleo*), regions upstream and downstream of *comK* were amplified with primer pairs PG211-PG212 and PG213-PG214, and subsequently digested with *Nco*I or *Bam*HI, respectively. A phleomycin resistance cassette was amplified from plasmid pIC22 [63] with primers PG215 and PG216, and digested with the corresponding restriction enzymes prior to ligation. Mutants were verified by PCR and by checking the loss of transformability. To construct strain PG455 (*aprE*::*spc PcomG-comK*), the *PcomG-comK* region, including the *spc* resistance cassette, was amplified from chromosomal DNA of strain PG401 with primers PG269 and PG270. Next, 2 kb fragments comprising the 5’ or 3’ half of the *aprE* gene were amplified with primer pairs PG271-PG272 and PG273-PG274, respectively. The three fragments were digested with *Bam*HI, ligated and transformed to strain BSB1. Integration was verified with PCR and by sequencing. To construct strain PG479 (*kre*::*erm*), regions upstream and downstream of *kre* were amplified with primer pairs PG306-PG307 and PG308-PG309, and subsequently digested with *Bam*HI and *Nco*I, respectively. An erythromycin resistance cassette was amplified from pMutin4 [58] with primer pair PG312-PG313, and digested with the corresponding enzymes prior to ligation.

### Transposon mutagenesis screen

Random transposon mutagenesis of strain PG401 (*PcomG-comK*, Δ*mecA*, *PcomG-lacZ-gfp*) was carried out using the mariner transposable element TnYLB-1 [35]. PG401 is not transformable, therefore plasmid pMarB was introduced by protoplast transformation using standard protocols. Individual colonies carrying the transposon plasmid were picked and grown in LB at 30°C for 6 h. Aliquots were frozen and stored at -80°C. Serial dilutions of each culture were plated on Nutrient agar plates containing kanamycin or erythromycin and incubated at 50°C overnight to induce transposition. The following day, the clone with the highest ratio of kan^R^/erm^R^ colonies, indicative of efficient transposition [35], was chosen for further experiments. An aliquote of the selected clone was diluted and plated on Nutrient agar plates, and incubated at 50°C to construct a library of approximately 45,000 transposon colonies. The colonies were then scraped off the plates, aliquoted and frozen. About 30,000 clones of the library were plated on Nutrient agar plates supplemented with 160 μg/ml X-gal and incubated at 37°C for 24 hours. Colonies showing an intense blue colour were reisolated, checked for integration of the transposon (kan^R^) and loss of the plasmid (erm^S^), and inspected by fluorescence microscopy to assess the frequency of GFP-expressing cells. Two rounds of backcrosses were performed: First, chromosomal DNA of the selected mutant strains was transformed into strain PG389 (*PcomG-lacZ-gfp*). The resulting strains were transformed with chromosomal DNA of PG401 so to introduce simultaneously the *PcomG-comK* and Δ*mecA* mutations and reconstitute the artificial ComK feedback loop. Chromosomal DNA from colonies that still showed an increase frequency of competent cells on nutrient agar was then re-introduced into PG401 by SPP1 phage transduction [64]. Transposon insertions were located by arbitrary PCR followed by sequencing.

### Microscopic imaging and GFP measurements

Cells were mounted on microscope slides coated with a thin layer of 1.2% agarose. Images were acquired with a Zeiss Axiovert 200M or a Nikon T1 microscope coupled to a Sony Cool-Snap HQ cooled CCD camera (Roper Scientific), and using Metamorph imaging software (Universal Imaging). Images were analysed and prepared for publication with ImageJ [65].

Initially (S2 Table and Figs 3, 8, S1, S2, and S6), GFP intensities of individual cells were measured manually with ImageJ [65], and subtracted by background GFP intensity levels, measured for each image. In later experiments (Figs 4 and 5), an in house developed ImageJ plugin (NucTracer (Syvertsson and Hamoen)) was used to semi-automatically determine cellular GFP levels. NucTracer, which uses nucleoids as region of interest (ROI) to measure GFP intensities, was employed to determine the GFP-ComK signals in Fig 4B. NucTracer was also used in Fig 5A and 5B, but here nucleoids were outlined with DAPI staining. To this end, the *Pxyl-gfp* and *Physp-gfp* reporter containing cells were grown in LB at 37°C in the presence or absence of 0.1% xylose or 50 μM IPTG, respectively. When cultures reached O.D._600_ of ~0.2, aliquots were concentrated 4 times in PBS supplemented with 2 μg/ml DAPI and transferred onto microscope slides.

To determine the fraction of ComK expressing cells using the *PcomG*-GFP reporter, a threshold value, generally 100 or 200 A.U., was used to separate cells in expressing and non-expressing categories. This threshold value is well above (~ 3 to 5 times) the fluorescent level of wild type (non GFP-expressing) cells.

### Luciferase assay

Overnight cultures in competence medium were diluted 20 fold in fresh medium and grown at 37°C until OD_600_ 0.1, then diluted 10 fold and 150 μl distributed into a black 96-well plate. Beetle Luciferin (Potassium salt, Promega) was added to a final concentration of 1.5 mg/ml (4.7 mM), and the cultures were incubated at 37°C in a FluoStar Optima plate reader (BMG-LabTech). Relative luminescence units (R.L.U.) and OD_600_ were measured with 10 min time intervals.

### Transformation of competent *B*. *subtilis* strains

Transformation of competent *B*. *subtilis* cells was performed using a two-step starvation procedure [4,49]. Briefly, overnight cultures were diluted 10 fold in 10 ml competence medium and incubated at 37°C under vigorous shaking. After 3 hours of growth, an equal volume of prewarmed “starvation medium” (competence medium lacking tryptophan, Cas aminoacids and ferric ammonium citrate) was added and incubation was continued for another 2 hours, prior to DNA addition. DNA was added to 400 μl aliquots, and incubation was prolonged for 1 hour at 37°C prior to plating onto selective nutrient agar plates.

### DNA transformation frequency assay

Transformation frequency was determined by transforming competent cultures with genomic DNA carrying an antibiotic resistance marker. To test the transformation frequency of a *kre* mutant compared to the wild type strain BSB1, exponentially growing cultures were diluted to OD_600_ ~ 0.01 in warm competence medium and grown at 37°C. The optical density of the cultures was measured at regular intervals. At the time of transition to stationary phase (T_0_), as well as 1 and 2 hours afterwards, DNA was added to 400 μl aliquots to a final concentration of 2.5 μg/ml, and incubation was prolonged for 1 hour at 37°C. Serial dilutions were plated on selective and unselective LB plates respectively. Transformation frequencies were calculated as 100 x (transformants/ml / CFU/ml). Relative transformation frequencies were normalized to the frequency of wild type strain. To test the transformation frequency upon *kre* overexpression, overnight cultures were diluted 10 fold in the presence or in the absence of 1 mM IPTG and the protocol used for routine transformations was followed as described in the previous section. DNA was added at a final concentration of 2 μg/ml.

### Western blotting

Exponentially growing cultures were diluted to OD_600_ ~ 0.01 in warm competence medium and incubated at 37°C. Optical density was measured at regular intervals and 1 ml samples were collected, spun down and flash frozen in liquid nitrogen at the time of transition to stationary phase (T_0_) and 1, 2, 4 hours after that time point. Incubation was prolonged overnight and one last sample (T_on_) was collected the following morning. Cell pellets were resuspended in 100 μl of lysis buffer (100 mM Tris-Cl pH 7.5, 2 mM EDTA, supplemented with Roche Complete mini protease inhibitor) containing 10 μg/ml lysozyme, incubated 10 min at 37°C and then sonicated. Cell debris were removed by centrifugation. Relative protein concentrations were estimated with a Bio-Rad protein assay and equal amount of proteins were loaded on NuPAGE 4–12% Bis-Tris gradient gels which were run in MES buffer (Life Technologies). Proteins were transferred onto a Hybond-P PVDF membrane (GE Healthcare) by using a wet procedure and western blotting was performed according to standard methods. A 1:5,000 dilution of rabbit polyclonal anti-ComK serum was used. Anti-rabbit horseradish peroxidase-linked antiserum (Sigma) was used as secondary antibody at a dilution of 1:10,000. Protein bands were detected using an ImageQuant LAS 4000 mini digital imaging system (GE Healthcare).

### Flow cytometry

Overnight cultures grown at 37°C in fructose-based competence medium were washed in 0.2 μM filtered starvation medium, stained with the red-fluorescent membrane dye FM5-95, diluted 300 fold in filtered starvation medium and directly analyzed on a CyFlow Space flow cytometer (Partec). Cell particles were selected based on the red-fluorescent signal. For each sample, 200,000 cells were analyzed and GFP signals were collected. Data were captured using FlowMax software (Quantum Analysis GmbH) and further analyzed using Cyflogic software (http://www.cyflogic.com), which was also used for graph preparation.

### β-galactosidase activity assay

β-galactosidase assays were performed in exponentially growing cultures as described by Daniel *et al*. [66] and the units of enzymatic activity calculated as described by Miller [67].

### Transcriptome experiment

To analyse the differences in transcriptome expression between wild-type *B*. *subtilis* (strain 168) and the *kre* mutant (PG479), microarray analyses were performed using an 8x15k Custom Agilent microarray. The NCBI annotation BSU41030 *B*. *subtilis* subsp. *subtilis* str. 168, complete genome, 2006-05-02 GenBank, containing information for 4105 transcripts, was used to design three probes per transcript. To isolate RNA, cell pellets were flash frozen in liquid nitrogen immediately after harvesting and stored at -80°C. Frozen pellets were grounded and subjected to RNA extraction as described previously [68], yielding RIN values of ≥ 9.6. Labeling was performed by reverse transcription using random octamers, incorporating Cy3 for the test samples and Cy5 for the common reference, as described [69]. The common reference was a pool of equal amounts of total RNA taken from all test samples. Hybridization, washing, and scanning was performed as described in the Two-Color Microarray-Based Gene Expression Analysis manual (Version 6.6, Agilent Technologies). Briefly, hybridization mixtures were made by combining 300 ng test (Cy3) and 300 ng common reference (Cy5) material and were subsequently hybridized to the Agilent SurePrint Custom 8x15k microarrays G2509F (Agilent Technologies). Two biological replicates were used for strain 168, while three biological replicates were used for strain PG479. The raw and normalized data from all arrays were subjected to various quality control checks [68]. Normalized expression values were calculated by using the robust multi-array average (RMA) algorithm [70], collecting and summarizing the intensity values of probes associated with a specific BSU locus tag. Differences in gene expression between wild-type and the *kre* mutant strain (PG479) were statistically analysed using the Limma package in R 2.14.1 (http://cran.r-project.org/). Empirical Bayes test statistics were used for calculating P-values [71], and for calculating false discovery rate corrected P-values [72]. Gene expression data and array design have been deposited at the public repository Gene Expression Omnibus, accession number GSE61757.

### Quantitative real-time PCR (qPCR)

Cultures were grown in LB at 37°C and, at O.D._600_ ~0.25, 5 ml volumes were spun at 6,000 rpm for 4 min and flash frozen in liquid nitrogen. Samples were processed with FastRNA Pro kit (MP Biomedicals), cell disruption was achieved by shaking samples 4 times per 20 seconds at 6,000 rpm in a Precellys24 Tissue homogenizer (Bertin technologies). RNA was further purified with Qiagen RNeasy kit. Total RNA (0.2 μg) was retro-transcribed using Multiscribe reverse transcriptase and a High-Capacity cDNA reverse transcription kit (Applied Biosystems). cDNA samples were diluted 1:24 and 6 μl was added to 10 μl GoTaq qPCR Master Mix (Promega) and 2 μl of each primer stock (final concentration of 0.5 μM for each primer). qPCR was performed on a Rotor-Gene Q cycler (QIAGEN) with 40 cycles of 5 s at 95°C and 10 s at 60°C. Cycle threshold (C_T_) values were obtained according to the software instructions. Relative quantification was performed with the 2^-ΔΔC^_T_ method [73]. *pfkA* mRNA levels were used as normalizer in Fig 6A. Changes in expression given are the average of 3 biological replicates, and the differences were statistically tested using an ANOVA model with coefficients for strain and replicate batch [74]. Oligonucleotides pairs used for qPCR were PG475-PG476 (*pfkA*), PG456-PG474 (*veg*), PG466-PG486 (*pksA*), PG495-PG496 (*ftsZ*), PG489-PG490 (*comK*) and PG471-PG472 (*kre*), and their sequences are listed in S3 Table.

### mRNA stability assay

Strains PG500 (*amyE*::*Pveg-lacZ-gfp*), PG512 (*amyE*::*Pveg-lacZ-gfp*, Δ*kre*) and PG474 (*amyE*::*Physp*-*kre*) were grown in LB at 37°C. At O.D.600 of ~0.2, T0 samples were collected (1 ml) and rifampicin added to a final concentration of 150 μg/ml. Samples were taken at time intervals (minutes) after rifampicin addition, and immediately stabilized by mixing them with equal volumes of RNAlater solution (Ambion). RNA was isolated and quantified using qPCR. Abundance of *comK* and *ftsZ* transcripts relative to the T0 sample was calculated with the 2^ΔC^_T_ equation, and average values and standard deviations were calculated from 3 biological replicates. mRNA half-lives were determined from an exponential fit to a plot of relative mRNA abundance versus time. The logit transformed relative mRNA abundances were subjected to an ANOVA, to test for differences at each time point. The p-values were corrected for false discoveries using Benjamini-Hochberg correction. Calculations were carried out using Microsoft Excel and R statistical software (http://cran.r-project.org/).

## Supporting Information

S1 Fig Transposon insertion in *ykyB* increases activation of the artificial ComK feedback loop. Strains PG401 (*amyE*::*PcomG-lacZ-gfp*, *PcomG-comK*, Δ*mecA*) and PG401-Tn4 (*amyE*::*PcomG-lacZ-gfp*, *PcomG-comK*, Δ*mecA*, *ykyB*:*Tn*) were grown overnight at 37°C on competence medium plates. Cells from plates were imaged by fluorescent light microscopy. (A) Average GFP intensity in cells of a representative experiment. Approximately 150 cells were measured for each strain. (B) Fraction of *PcomG* ‘ON’ (ComK expressing) cells. Cells were considered ‘ON’ when the GFP intensity exceeded a threshold of 200 A.U. (C) Inactivation of *ykyB* is responsible for the activation of the ComK loop in PG401 background. Strains PG401 and PG539 (*amyE*::*PcomG-lacZ-gfp*, *PcomG-comK*, Δ*mecA*, Δ*ykyB*) were grown overnight in liquid LB medium at 37°C and imaged by fluorescent light microscopy. Phase contrast, GFP images and arbitrary GFP colour intensity scales are shown.
(TIF)

S2 Fig Effect of *kre* deletion on different competence reporter fusions. Strains PG389 (*amyE*::*PcomG-lacZ-gfp*), PG433 (*amyE*::*PcomG-lacZ-gfp*, *kre*:*Tn*), PG435 (*amyE*::*PcomC-lacZ-gfp*), PG448 (*amyE*::*PcomC-lacZ-gfp*, *kre*:*Tn*), PG436 (*amyE*::*PcomF-lacZ-gfp*), PG449 (*amyE*::*PcomF-lacZ-gfp*, *kre*:*Tn*), PG437 (*amyE*::*PaddAB-lacZ-gfp*), PG450 (*amyE*::*PaddAB-lacZ-gfp*, *kre*:*Tn*), PG438 (*amyE*::*PnucA-lacZ-gfp*) and PG457 (*amyE*::*PnucA-lacZ-gfp*, *kre*:*Tn*) were grown on competence medium plates. Microscopy images were taken after overnight incubation at 37°C. The average amounts of GFP per cell was measured in at least 300 cells, and cells were counted as competent when average GFP intensity exceeded 100 A.U. Frequency of competent cells for each promoter fusion in wild-type (left, gray columns) and *kre* mutant background (right, red columns) is shown for 3 independent experiments.
(TIF)

S3 Fig DNA transformation frequency after *kre* overexpression. Strains PG474 (*amyE*::*Physp-kre*) and PG548 (*amyE*::Physp-kre*) were grown in competence medium in the presence or absence of 1 mM IPTG. PG548 contains a frame shift mutation in the start codon of *kre* (kre*). Average and standard deviation of 2 independent experiments are shown.
(TIF)

S4 Fig Map of the *ykyB* (*kre*) transcription locus. Image was taken from the *B*. *subtilis* expression data browser (http://genome.jouy.inra.fr/cgi-bin/seb/index.py) based on data from [38]. (I) Genbank annotation, (II) upshifts, downshifts and transcription units, (III) transcription profiles of both DNA strands across different conditions. See [38] for detailed explanations.
(TIF)

S5 Fig Translational GFP fusions with Kre. Strains PG442 (*amyE*::*Pxyl-msfGFP-kre*) (A) and PG494 (*amyE*::*Pxyl-kre-msfGFP*) (B) were grown at 37°C in fructose-based competence medium in the presence of 1% xylose. GFP and phase contrast images were taken 1 hour after the point of transition to stationary phase.
(TIF)

S6 Fig Functionality of a GFP-Kre translational fusion. Flow cytometric analysis of *comG-gfp* expression in fructose-based competence medium. Induction of GFP-Kre reduces GFP expression from the *comG* promoter indicating that the fusion protein is active. Strain were grown overnight at 37°C with and without 1% xylose. 200,000 cells were analysed for each strain, as described. Representative graphs are shown for each set of strains. (A) Analysis of strains PG342 (*comG-gfp*) and PG482 (*amyE*::*Pxyl-msfGFP-kre*, *comG-gfp*). (B) Analysis of strains PG425 (*comG-gfp*, *kre*:Tn) and PG485 (*amyE*::*Pxyl-msfGFP-kre*, *kre*:Tn, *comG-gfp)*. (C) Control graph showing that GFP-Kre signal does not interfere with the *comG-gfp* one. The wild type strain BSB1 and strain PG442 (*amyE*::*Pxyl-msfGFP-kre*) were grown overnight in the presence of 1% xylose. (D) Control graph showing the reduction of *comG-gfp* expressing cells upon Kre overexpression. Strain PG342 (*comG-gfp*) and PG490 (*amyE*::*Physp-kre*, *comG-gfp*) were grown overnight in the presence or in the absence of 1 mM IPTG.
(TIF)

S7 Fig Kre activity is not *comK* locus dependent. (A) Antisense S365 transcript overlapping the *comK* locus. (I) Genbank annotation and (II) the new annotation of transcription segments taken from the *B*. *subtilis* expression data browser (http://genome.jouy.inra.fr/cgi-bin/seb/index.py) [38]. (B) Representative microscopic images of PG461 (*aprE*::*PcomG-comK*, Δ*mecA*, Δ*comK*, *amyE*::*PcomG-lacZ-gfp*) and PG463 (*aprE*::*PcomG-comK*, Δ*mecA*, Δ*comK*, *amyE*::*PcomG-lacZ-gfp*, *kre*:*Tn*), grown on competence medium plates. GFP images are shown with the same contrast settings and coloured with a colour-intensity scale. Lower panels show related phase contrast images. (C) Average GFP intensity per cell measured in PG461 and PG463. (D) Average GFP intensity per cell measured in PG401 and PG401-Tn4.
(TIF)

S8 Fig Expression of *Pkre-gfp* in a Δ*comK* background. Strains PG604 (*kre*:*Pkre-gfp*) and PG678 (*kre*:*Pkre-gfp*, *ΔcomK*) were grown at 37°C in competence medium supplemented with 5 μg/ml chloramphenicol. Phase contrast and GFP images were taken after overnight incubation. GFP levels are shown with an arbitrary colour intensity scale.
(TIF)

S9 Fig Induction of *kre* during competence reduces transformation frequency. Wild type (wt) cells (PG746), and cells containing *kre* expressed by *PcomG* driven expressing with (PG753) or without the native *kre* gene (PG755) were grown in competence medium. The transformation efficiencies were calculated relative to PG746 (wt). Average and standard deviation of two independent experiments are shown.
(TIF)

S1 Table Standard errors and p-values for mRNA stability measurement of Fig 6. (A) half-life estimates and their standard errors for each individual mRNA stability experiments upon which Fig 6B–6E are based. (B) p-values and false discovery rate corrected p-values indicating the significance of the difference in mRNA abundance at each time point in Fig 6B–6E.
(PDF)

S2 Table Effect of glucose on the artificial ComK feedback loop. Strain PG401 (*amyE*::*PcomG-lacZ-gfp*, *PcomG-comK*, Δ*mecA*) was grown in LB in the presence or in the absence of 0.5% glucose. Prior to microscopic imaging, cells were briefly incubated with FM5-95 to stain membranes, in order to allow single cell detection. GFP, phase contrast and FM5-95 images were taken during logarithmic growth (LOG) and two hours after the transition to stationary phase (STAT). Cells were counted as competent (ComK expressing) when the GFP intensity exceeded 200 A.U., and the result of two independent experiments are shown. Number of cells analysed are indicated between brackets.
(PDF)

S3 Table Oligonucleotides used in this study. (PDF)
