# Supplementary figures and images for: A Novel Feedback Loop That Controls Bimodal Expression of Genetic Competence
Source: PLoS Genet. 2015 Jun 25;11(6):e1005047. doi: 10.1371/journal.pgen.1005047 (PMC4482431; doi:10.1371/journal.pgen.1005047)

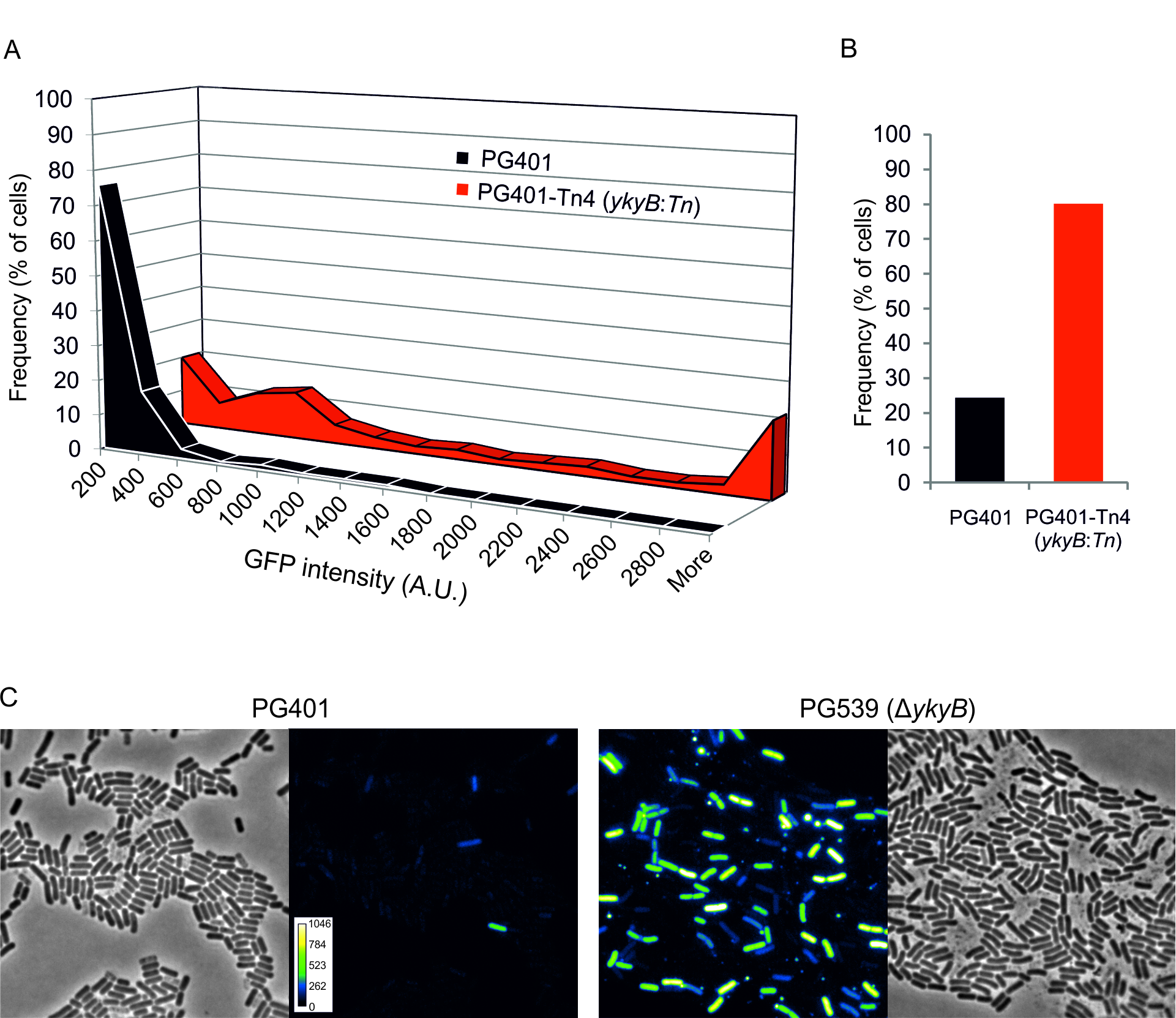

Supplement: S1 Fig — Strains PG401 (amyE::PcomG-lacZ-gfp, PcomG-comK, ΔmecA) and PG401-Tn4 (amyE::PcomG-lacZ-gfp, PcomG-comK, ΔmecA, ykyB:Tn) were grown overnight at 37°C on competence medium plates. Cells from plates were imaged by fluorescent light microscopy. (A) Average GFP intensity in cells of a representative experiment. Approximately 150 cells were measured for each strain. (B) Fraction of PcomG ‘ON’ (ComK expressing) cells. Cells were considered ‘ON’ when the GFP intensity exceeded a threshold of 200 A.U. (C) Inactivation of ykyB is responsible for the activation of the ComK loop in PG401 background. Strains PG401 and PG539 (amyE::PcomG-lacZ-gfp, PcomG-comK, ΔmecA, ΔykyB) were grown overnight in liquid LB medium at 37°C and imaged by fluorescent light microscopy. Phase contrast, GFP images and arbitrary GFP colour intensity scales are shown. (TIF) [file pgen.1005047.s001.tif]

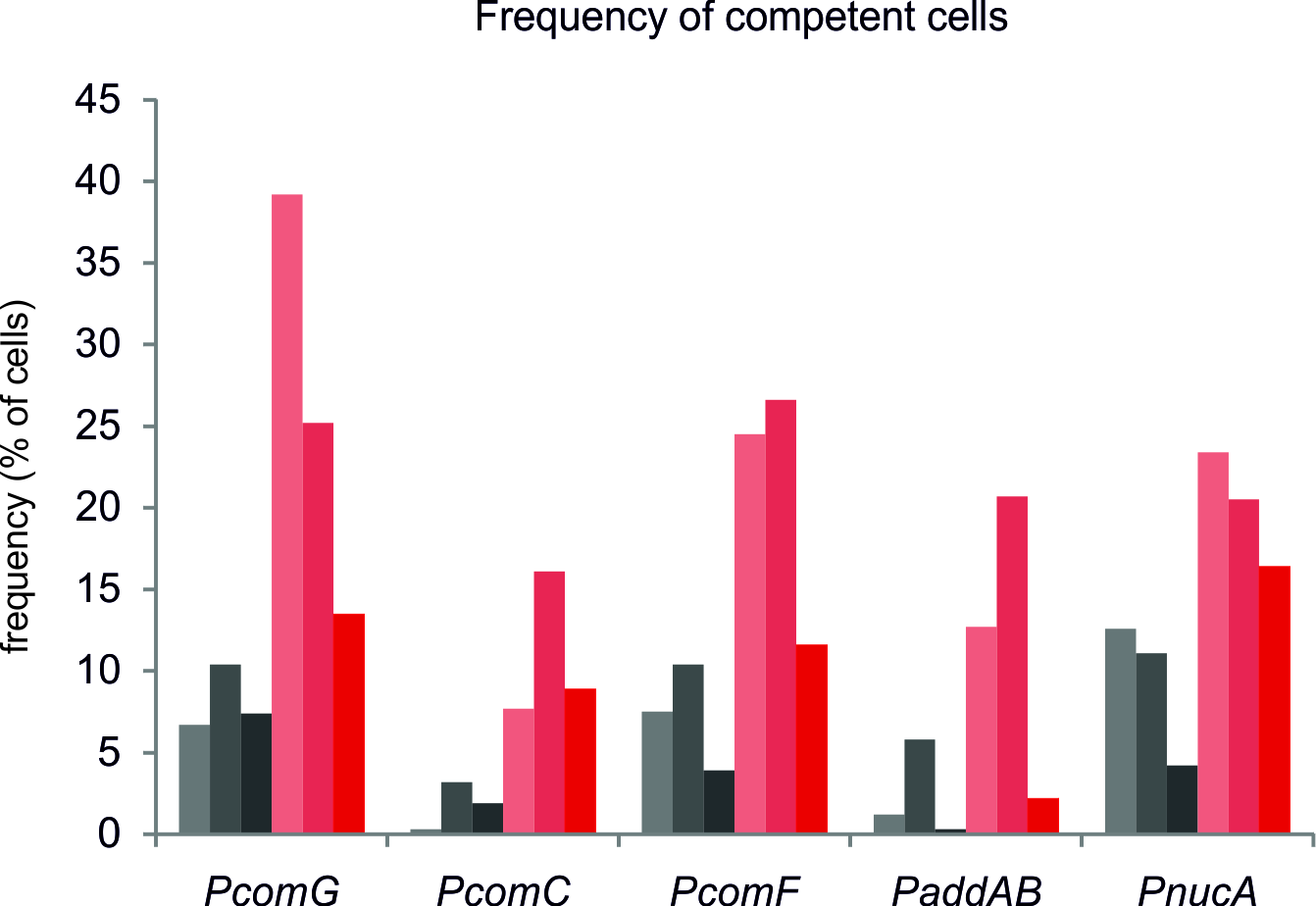

Supplement: S2 Fig — Strains PG389 (amyE::PcomG-lacZ-gfp), PG433 (amyE::PcomG-lacZ-gfp, kre:Tn), PG435 (amyE::PcomC-lacZ-gfp), PG448 (amyE::PcomC-lacZ-gfp, kre:Tn), PG436 (amyE::PcomF-lacZ-gfp), PG449 (amyE::PcomF-lacZ-gfp, kre:Tn), PG437 (amyE::PaddAB-lacZ-gfp), PG450 (amyE::PaddAB-lacZ-gfp, kre:Tn), PG438 (amyE::PnucA-lacZ-gfp) and PG457 (amyE::PnucA-lacZ-gfp, kre:Tn) were grown on competence medium plates. Microscopy images were taken after overnight incubation at 37°C. The average amounts of GFP per cell was measured in at least 300 cells, and cells were counted as competent when average GFP intensity exceeded 100 A.U. Frequency of competent cells for each promoter fusion in wild-type (left, gray columns) and kre mutant background (right, red columns) is shown for 3 independent experiments. (TIF) [file pgen.1005047.s002.tif]

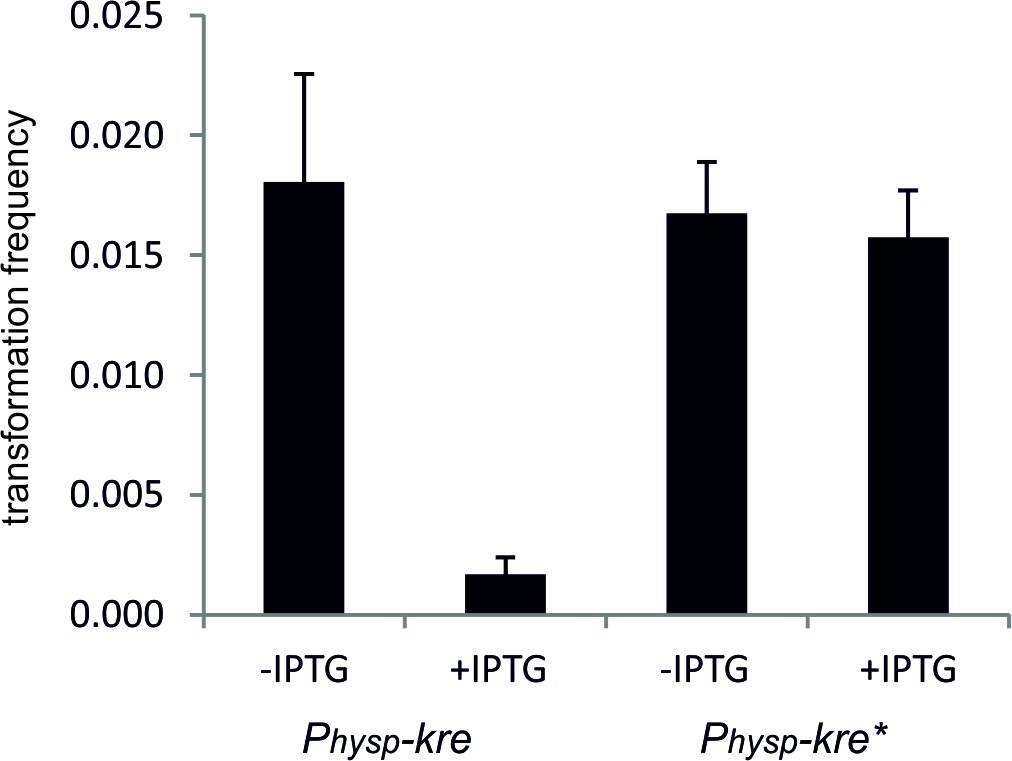

Supplement: S3 Fig — Strains PG474 (amyE::Physp-kre) and PG548 (amyE::Physp-kre*) were grown in competence medium in the presence or absence of 1 mM IPTG. PG548 contains a frame shift mutation in the start codon of kre (kre*). Average and standard deviation of 2 independent experiments are shown. (TIF) [file pgen.1005047.s003.tif]

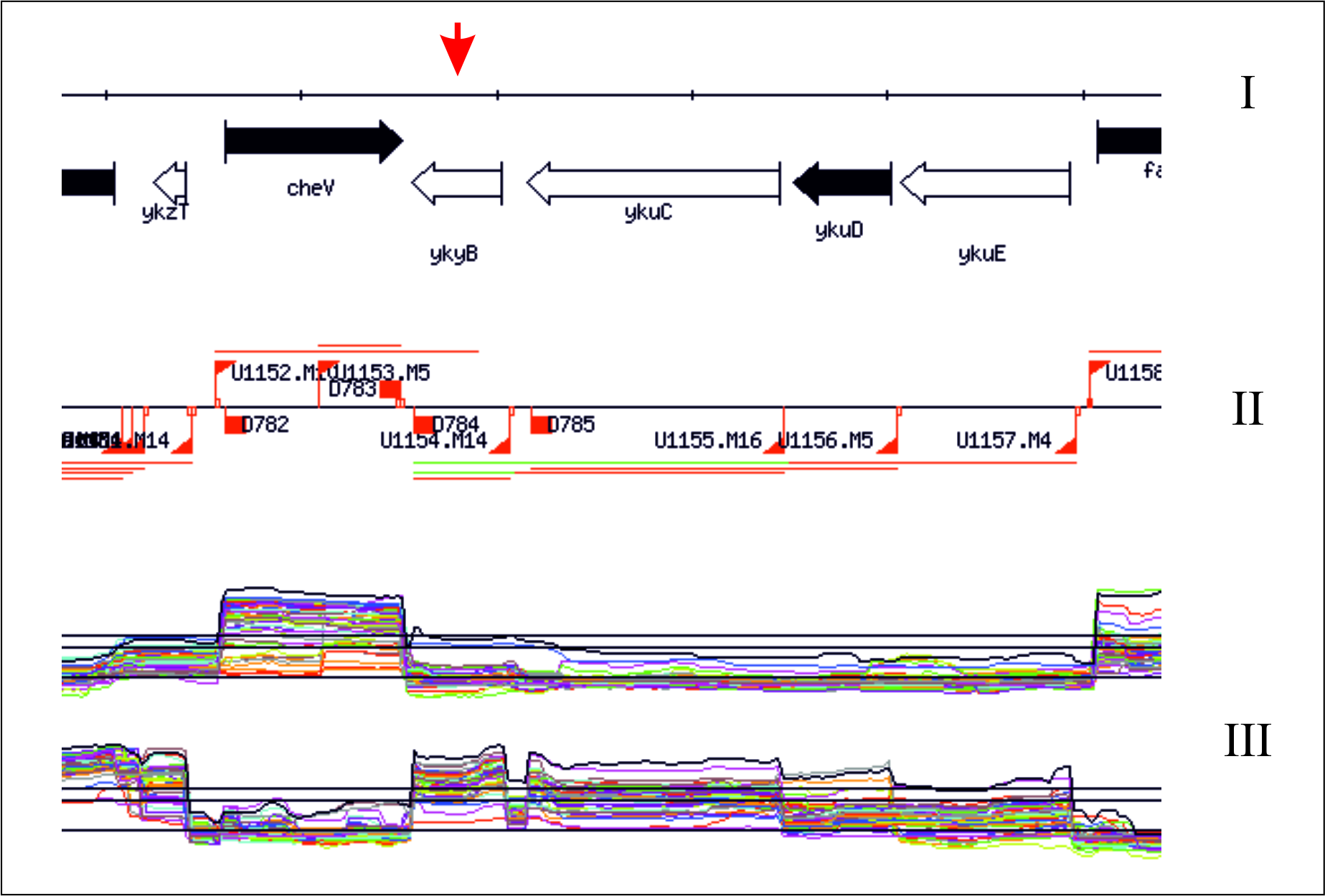

Supplement: S4 Fig — Image was taken from the B. subtilis expression data browser (http://genome.jouy.inra.fr/cgi-bin/seb/index.py) based on data from [38]. (I) Genbank annotation, (II) upshifts, downshifts and transcription units, (III) transcription profiles of both DNA strands across different conditions. See [38] for detailed explanations. (TIF) [file pgen.1005047.s004.tif]

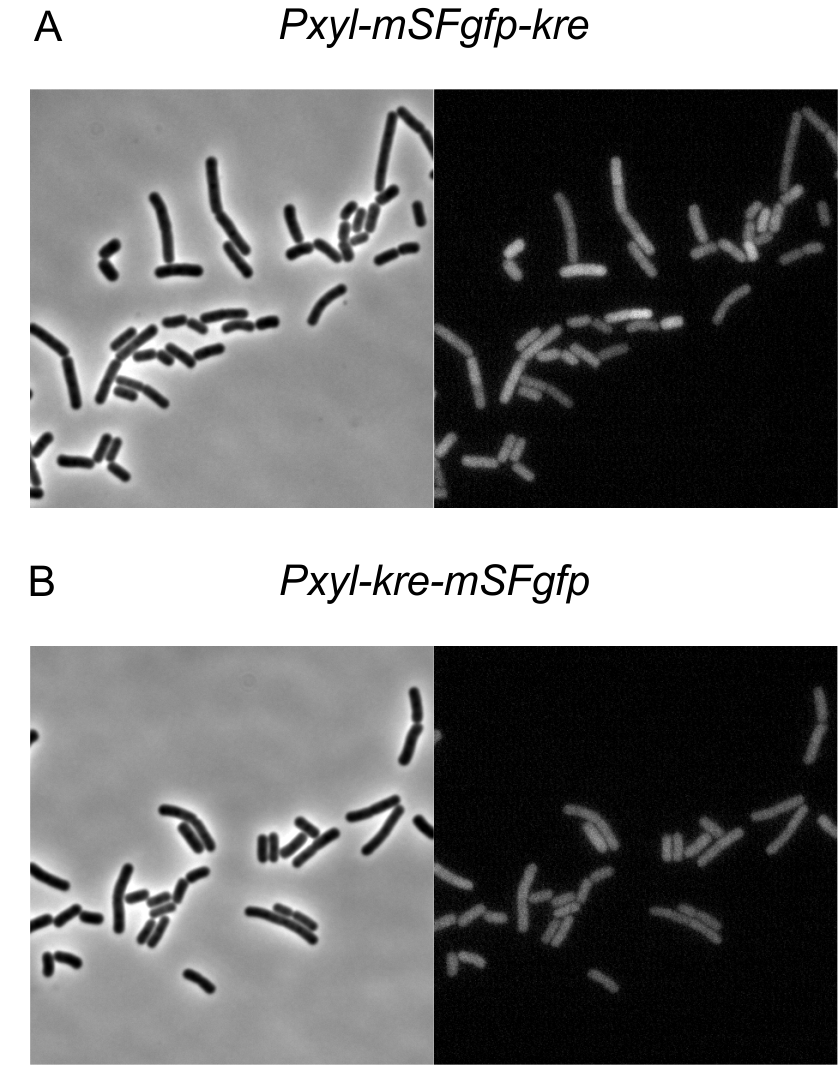

Supplement: S5 Fig — Strains PG442 (amyE::Pxyl-msfGFP-kre) (A) and PG494 (amyE::Pxyl-kre-msfGFP) (B) were grown at 37°C in fructose-based competence medium in the presence of 1% xylose. GFP and phase contrast images were taken 1 hour after the point of transition to stationary phase. (TIF) [file pgen.1005047.s005.tif]

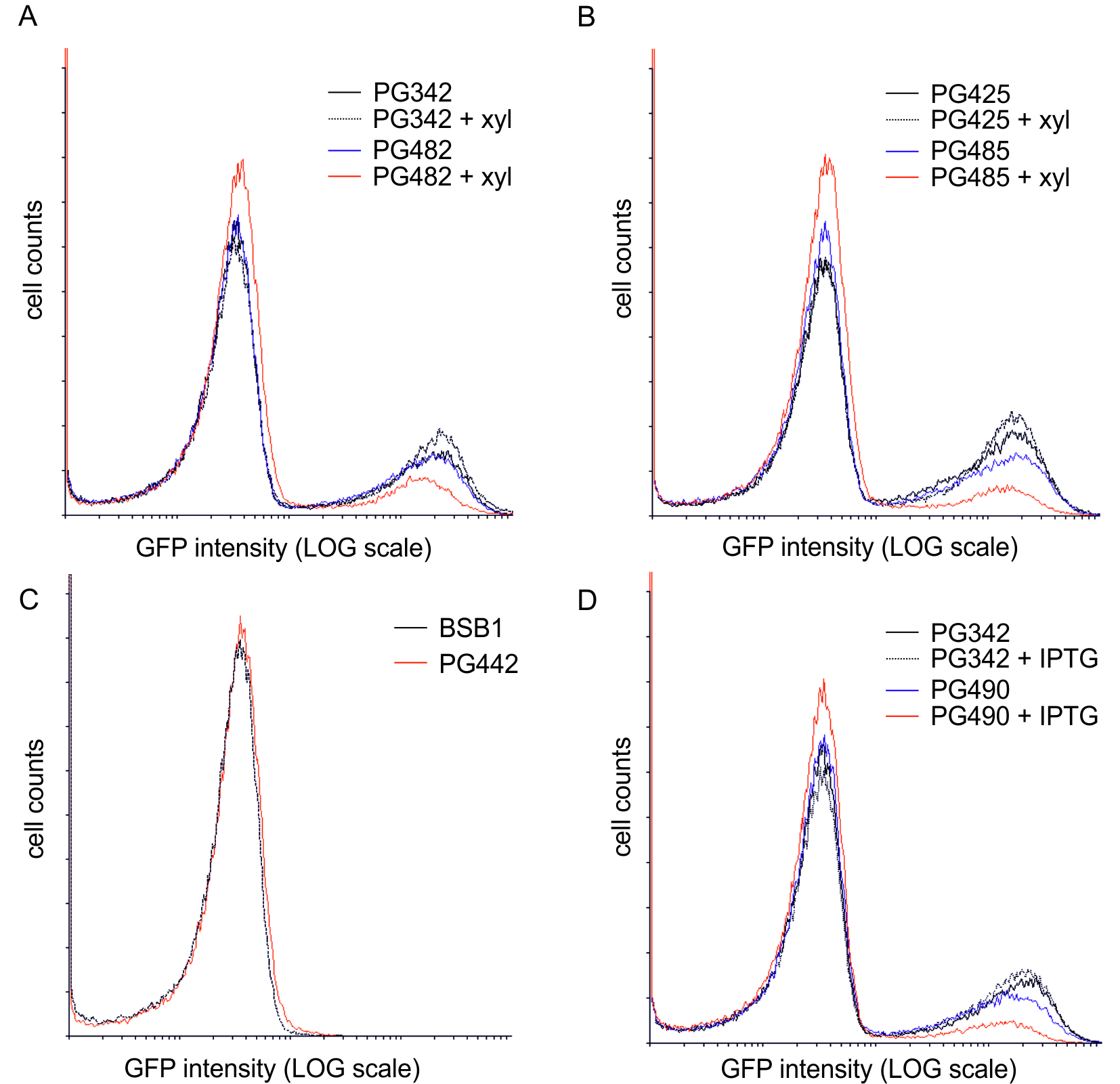

Supplement: S6 Fig — Flow cytometric analysis of comG-gfp expression in fructose-based competence medium. Induction of GFP-Kre reduces GFP expression from the comG promoter indicating that the fusion protein is active. Strain were grown overnight at 37°C with and without 1% xylose. 200,000 cells were analysed for each strain, as described. Representative graphs are shown for each set of strains. (A) Analysis of strains PG342 (comG-gfp) and PG482 (amyE::Pxyl-msfGFP-kre, comG-gfp). (B) Analysis of strains PG425 (comG-gfp, kre:Tn) and PG485 (amyE::Pxyl-msfGFP-kre, kre:Tn, comG-gfp). (C) Control graph showing that GFP-Kre signal does not interfere with the comG-gfp one. The wild type strain BSB1 and strain PG442 (amyE::Pxyl-msfGFP-kre) were grown overnight in the presence of 1% xylose. (D) Control graph showing the reduction of comG-gfp expressing cells upon Kre overexpression. Strain PG342 (comG-gfp) and PG490 (amyE::Physp-kre, comG-gfp) were grown overnight in the presence or in the absence of 1 mM IPTG. (TIF) [file pgen.1005047.s006.tif]

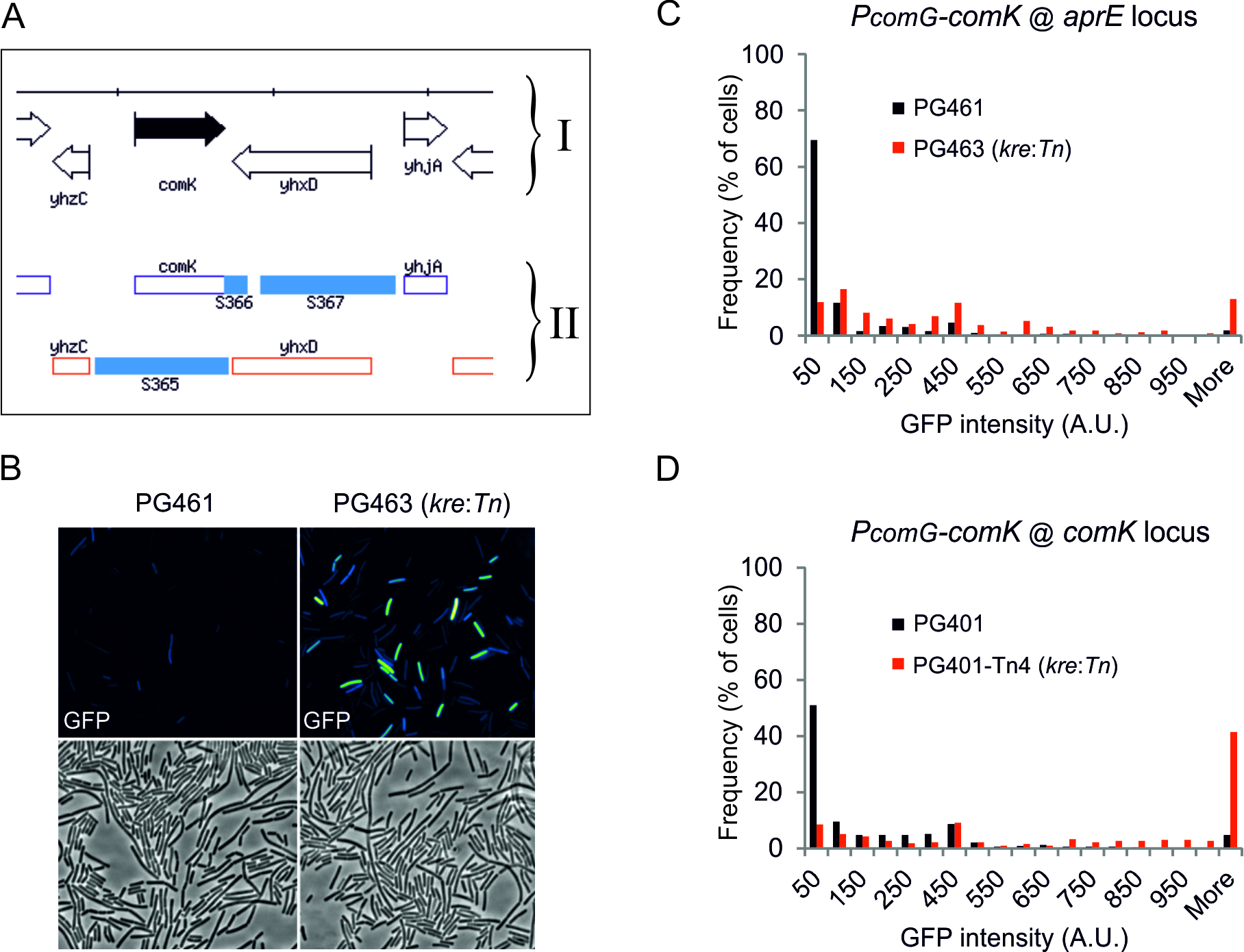

Supplement: S7 Fig — (A) Antisense S365 transcript overlapping the comK locus. (I) Genbank annotation and (II) the new annotation of transcription segments taken from the B. subtilis expression data browser (http://genome.jouy.inra.fr/cgi-bin/seb/index.py) [38]. (B) Representative microscopic images of PG461 (aprE::PcomG-comK, ΔmecA, ΔcomK, amyE::PcomG-lacZ-gfp) and PG463 (aprE::PcomG-comK, ΔmecA, ΔcomK, amyE::PcomG-lacZ-gfp, kre:Tn), grown on competence medium plates. GFP images are shown with the same contrast settings and coloured with a colour-intensity scale. Lower panels show related phase contrast images. (C) Average GFP intensity per cell measured in PG461 and PG463. (D) Average GFP intensity per cell measured in PG401 and PG401-Tn4. (TIF) [file pgen.1005047.s007.tif]

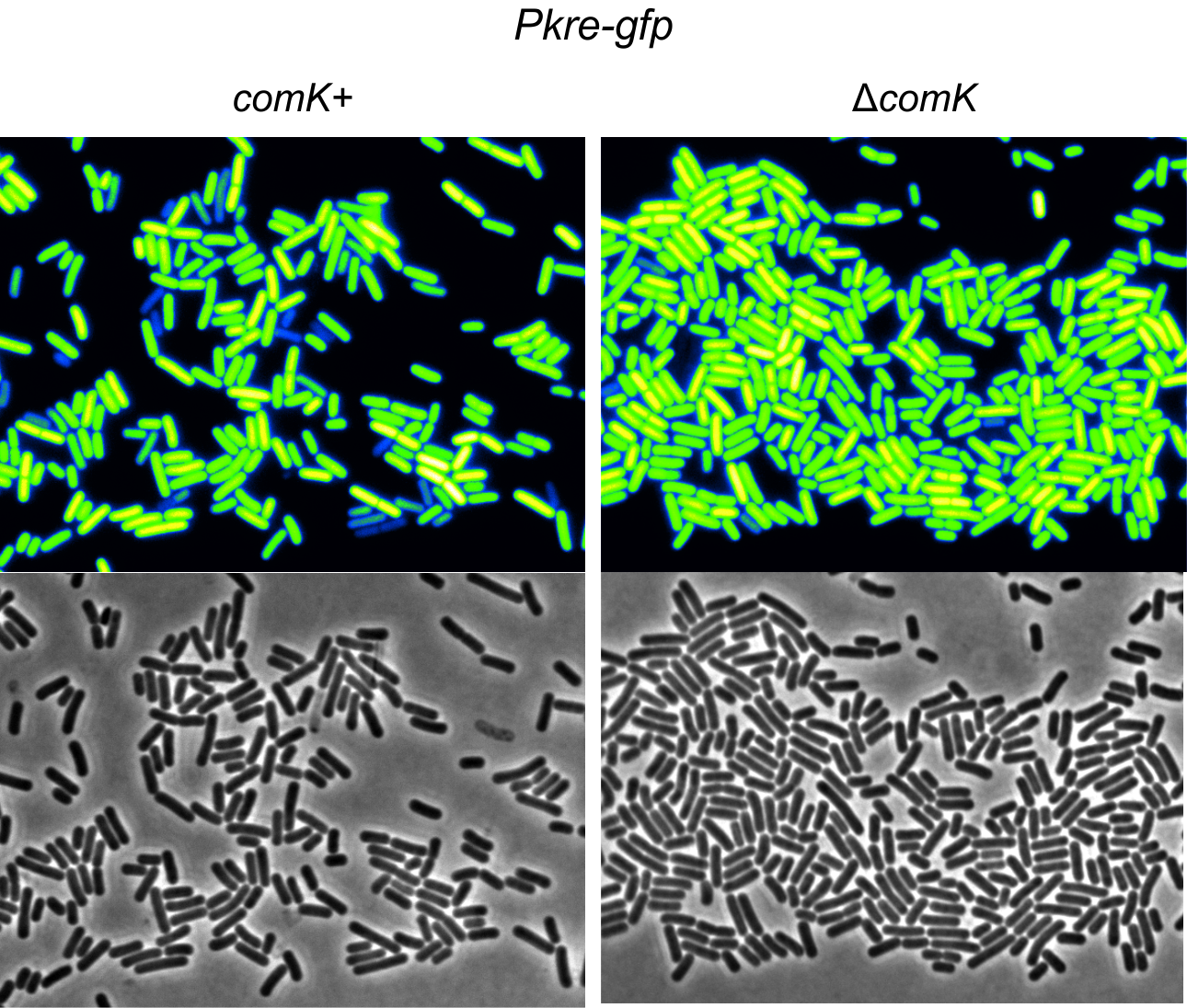

Supplement: S8 Fig — Strains PG604 (kre:Pkre-gfp) and PG678 (kre:Pkre-gfp, ΔcomK) were grown at 37°C in competence medium supplemented with 5 μg/ml chloramphenicol. Phase contrast and GFP images were taken after overnight incubation. GFP levels are shown with an arbitrary colour intensity scale. (TIF) [file pgen.1005047.s008.tif]

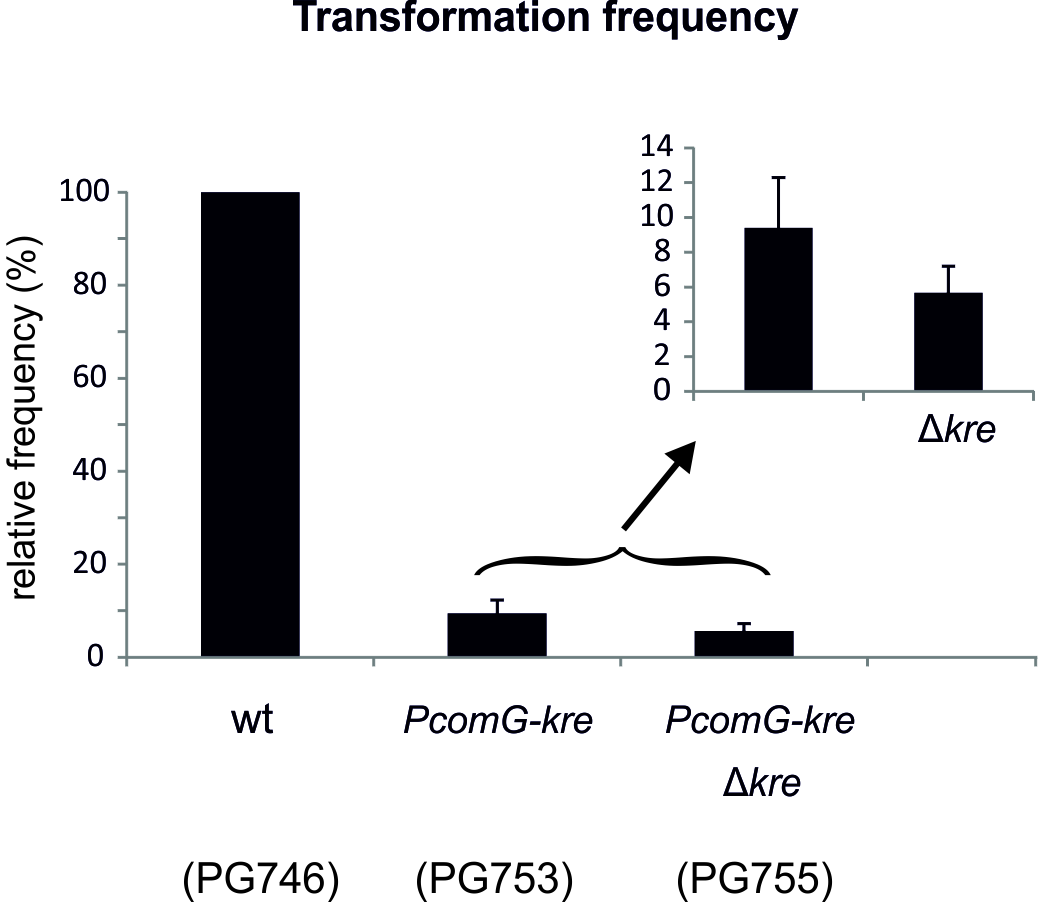

Supplement: S9 Fig — Wild type (wt) cells (PG746), and cells containing kre expressed by PcomG driven expressing with (PG753) or without the native kre gene (PG755) were grown in competence medium. The transformation efficiencies were calculated relative to PG746 (wt). Average and standard deviation of two independent experiments are shown. (TIF) [file pgen.1005047.s009.tif]
