# Supplementary material for: A Novel Feedback Loop That Controls Bimodal Expression of Genetic Competence
Source: PLoS Genet. 2015 Jun 25;11(6):e1005047. doi: 10.1371/journal.pgen.1005047 (PMC4482431; doi:10.1371/journal.pgen.1005047)
Supplement: S1 Table — (A) half-life estimates and their standard errors for each individual mRNA stability experiments upon which Fig 6B–6E are based. (B) p-values and false discovery rate corrected p-values indicating the significance of the difference in mRNA abundance at each time point in Fig 6B–6E. (PDF) [file pgen.1005047.s010.pdf]

Table S1-A

	comK half-life		*ftsZ* half-life	
	Estimate	Std. Error	Estimate	Std. Error
PG500	4.36	0.43	2.21	0.06
PG500	3.63	0.44	2.16	0.07
PG500	3.60	0.40	2.17	0.02
PG512	5.78	0.87	2.32	0.10
PG512	5.26	0.37	2.30	0.09
PG512	5.08	0.44	2.16	0.13
-IPTG	2.67	0.34	2.13	0.04
-IPTG	3.17	0.39	2.06	0.05
-IPTG	1.86	0.29	1.65	0.09
+IPTG	1.20	0.16	2.15	0.05
+IPTG	1.65	0.22	2.36	0.10
+IPTG	1.17	0.17	1.87	0.07

Table S1-B

	comK		*ftsZ*	
	Δkre vs wt		Δkre vs wt	
Time point	p-value	fdr. cor. p-value	p-value	fdr. cor. p-value
2	0.000	0.000	0.019	0.094
4	0.471	0.471	0.369	0.462
6	0.003	0.009	0.809	0.809
8	0.013	0.021	0.329	0.462
16	0.094	0.117	0.149	0.372

	PG474 vs wt		PG474 vs wt	
Time point	p-value	fdr. cor. p-value	p-value	fdr. cor. p-value
1	0.039	0.039	0.179	0.536
2	0.037	0.039	0.132	0.536
3	0.005	0.011	0.796	0.786
4	0.008	0.011	0.485	0.786
6	0.006	0.011	0.773	0.786
8	0.008	0.011	0.566	0.786
