# Supplementary material for: A Novel Feedback Loop That Controls Bimodal Expression of Genetic Competence
Source: PLoS Genet. 2015 Jun 25;11(6):e1005047. doi: 10.1371/journal.pgen.1005047 (PMC4482431; doi:10.1371/journal.pgen.1005047)
Supplement: S2 Table — Strain PG401 (amyE::PcomG-lacZ-gfp, PcomG-comK, ΔmecA) was grown in LB in the presence or in the absence of 0.5% glucose. Prior to microscopic imaging, cells were briefly incubated with FM5-95 to stain membranes, in order to allow single cell detection. GFP, phase contrast and FM5-95 images were taken during logarithmic growth (LOG) and two hours after the transition to stationary phase (STAT). Cells were counted as competent (ComK expressing) when the GFP intensity exceeded 200 A.U., and the result of two independent experiments are shown. Number of cells analysed are indicated between brackets. (PDF) [file pgen.1005047.s011.pdf]

	**LB LOG**	**LB + glc LOG**	**LB STAT**	**LB + glc STAT**
1st	0.18% (552)	0.30% (676)	3.34% (569)	14.64% (649)
2nd	0.22% (455)	0.92% (653)	1.92% (626)	15.29% (883)
