# Supplementary material for: A Novel Feedback Loop That Controls Bimodal Expression of Genetic Competence
Source: PLoS Genet. 2015 Jun 25;11(6):e1005047. doi: 10.1371/journal.pgen.1005047 (PMC4482431; doi:10.1371/journal.pgen.1005047)
Supplement: S3 Table — (PDF) [file pgen.1005047.s012.pdf]

Name	Restriction site	Sequence (5' - 3')
PG187	*Pml*I-*Bgl*II-*Age*I	CTAG**CACGTGAGATCTACCGGT**TTCCGCTCTTGTGCTGTTAGG
PG188	*Pml*I-*Bln*I-*Sac*II	CATA**CACGTGCCTAGG**T**CCGCGG**TCGAGGGTACCAGCGCTATC
PG189	*Eag*I	ATAT**CGGCCG**GAAGGAGATATACATATGGTGAGCAAGGGCGAG
PG190	*Spe*I	CATG**ACTAGT**GCTCGAATTCATTATTACTTGTACAGCTCGTCCATG
PG195	*Apa*I	ACAT**GGGCCC**GTCGGATCTGGTAATGACTC
PG196	*Not*I	ATCA**GCGGCCGC**GAAATGATGACCTCGTTTCC
PG201	*Bgl*II	ATCT**AGATCT**TGATGCCGGAACTGAATTGG
PG202	*Bln*I	CAGT**CCTAGG**TTTGATTCCCTCTCCTTTCA
PG203	*Sac*II	ATCA**CCGCGG**TATAGGGAAAAGGTGGTGAA
PG204	*Kpn*I	AGTC**GGTACC**GCCTGCCCGGTTATTATTAT
PG209	*Sph*I	GACT**GCATGC**TTTCGCTACGCTCAAATCCT
PG210	*Xma*I	CAGT**CCCGGG**AAAAAGTACAGTCGGCATTA
PG211		CTTTCGGCCAGTTGTCTCAG
PG212	*Nco*I	CAGT**CCATGG**ATTATGGCCTCCATCCTTTT
PG213	*Bam*HI	GTCA**GGATCC**AAAAATAGGAAGGAGCTGAC
PG214		TATCATCGCCTACGGATGTC
PG215	*Nco*I	GACT**CCATGG**CGATGACCTCTAATAATTGT
PG216	*Bam*HI	GACT**GGATCC**TCACTCAAAAAATCTCCACC
PG221	*Bln*I	TCAT**CCTAGGT**TACATAATTCACGTTATTG
PG222	*Xho*I	CAGT**CTCGAG**ACGACGGCCAGTGAATTCCC
PG223	*Bln*I	GACT**CCTAGG**GCCAACCTTCCCTTCTATAT
PG224		GGAAACCATGCTGCAGGAAG
PG225	*Xho*I	CAGT**CTCGAG**CACTTTGCATCATAGCAAAC
PG226		GTAGCTGTGTACGGAATGTC
PG269		TACCGCAATTCCGCCGACAC
PG270	*Bam*HI	GACT**GGATCC**TATTGGCCGCTTCCTGTTG
PG271	*Bam*HI	CAGT**GGATCC**GGCGGACCTACTGGTTCTAC
PG272		CTTGCTGGCTGCCGTCAATC
PG273	*Bam*HI	ATCA**GGATCC**GCCAAGATGGCATA
PG274	*Bam*HI	CAGT**GGATCC**TGAAAGGAGCTGCCCTGTTC
PG279	*Sac*II	ATAT**CCGCGG**AGGATGGGTACCCTGCAGAT
PG280	*Spe*I	ACTG**ACTAGT**TCATTATTTGTAGAGCTCATCCATGC
PG282	*Sac*II	ATAT**CCGCGG**GCCAGAACCTGATCCAGAGCCTGAACCAAAGTAACTCTCGCCAAGTT
PG287	*Bam*HI	ATATGGATCCGCGGTAGCGGCTCTGGAATGGACGACCATGCATATAC
PG288	*Eco*RI	GACGGAATTCTTAAAAGTAACTCTCGCCAAG
PG289	*Bgl*II	GCAAA**AGATCT**CTATGATGCAAGTG
PG290	*Bln*I	ATAT**CCTAGG**CACCTTTACTCATTGAGTTC
PG291	*Bgl*II	AGTC**AGATCT**GTGCCTTTTGAGAAGATTCG
PG292	*Bln*I	ATGA**CCTAGG**ATAGCACGCCTCCTTTCGAA
PG293	*Bgl*II	TGAC**AGATCT**CGCCTGATTTCTTCAATCTG

PG294	*Bln*I	ATAT**CCTAGG**AAGACCCCTCTCTTTTTATC
PG295	*Bgl*II	AGTC**AGATCT**AACAGAGGAGCAATTTGCTG
PG296	*Bln*I	CAGT**CCTAGG**TCACCTCCCGCTTTTAAAAC
PG306		AATGTAGCAGGATGGGCTCC
PG307	*Bam*HI	CAGT**GGATCC**CCTTTGCTCTTATCAATTTC
PG308	*Nco*I	CTGA**CCATGG**AAAATAAAAACAGCCGTGCC
PG309		TAATGACAATCGTTTGATCC
PG312	*Bam*HI	ATAT**GGATCC**TCGTGTTCGTGCTGACTTGC
PG313	*Nco*I	ATGT**CCATGG**TCCTTGGAAGCTGTCAGTAG
PG317	*Bgl*II	CAGT**AGATCT**TTACGCCGTTTATGGAACGG
PG318	*Bln*I	ATGA**CCTAGG**TCCACCTCACTACATTTATTG
PG319	*Bln*I	CTGA**CCTAGG**TTGATAAGAGCAAAGGAGAG
PG320	*Bgl*II	GACT**AGATCT**GAAAAACTAAAAAAAATATTG
PG321	*Bln*I	CAGTCCTAGGAATCTCCTTTCTAG
PG322	*Age*I	AGTG**ACCGGT**GTAAGCGGCATGCAGGACCC
PG323	*Bln*I	AGAT**CCTAGG**TTCTCTCCTTTGCTCTTATC
PG330	*Xba*I	TCAG**TCTAGA**TGATGCCGGAACTGAATTGG
PG332		GCAAAGGAGAGAAATAATAGGACGACCATGCATATACG
PG333		CGTATATGCATGGTCGTCCTATTATTTCTCTCCTTTGC
PG334	*Kpn*I	ATAT**GGTACC**TTTGACACTCGGCTTAGTCG
PG336	*Pst*I	CGAT**CTGCAG**CATTATTTCTCTCCTTTGCTC
PG418	*Bam*HI	GACT**GGATCC**TTCAACGCATATTGTAGAAAAAG
PG419	*Hind*III	AGTC**AAGCTT**TGATGCCGGAACTGAATTGG
PG438	*Spe*I	ATCG**ACTAGT**TTAAAAGTAACTCTCGCCAAG
PG456		ATGGCGAAGACGTTGTCC
PG466		GCAAAGGAAGCAGGATTGTC
PG471		CGAACATCTTCCGCATTTAG
PG472		TCATGCCGACATATTCTTGC
PG474		CGAACGCTCAATCGTTTTTC
PG475		TGTTGTTATCGGTGGAGACG
PG476		AAATCAGTGCCCGGAATG
PG486		CGGTCCGTTACCTTTTCTTG
PG489		CGTGCTGCCAGAAGAAATAG
PG490		AAAAATCGGCAGCTTCTGTC
PG495		AGGTGCTGACATGGTATTCG
PG496		CACAACGCCGACTGTTAATG
PG509	*Bgl*II	AGTC**AGATCT**GCTGAGGCTCCGCGGTAAAG
PG510	*Bln*I	CTGA**CCTAGG**ATAGCTTTATTGTAACAAG
